# Temporal Trends in Lower Respiratory Infection Mortality in Ecuador, 2012–2022

**DOI:** 10.3390/tropicalmed11010021

**Published:** 2026-01-12

**Authors:** Reena Krishna, Luis Furuya-Kanamori, Harriet L. S. Lawford

**Affiliations:** 1 School of Public Health, The University of Queensland, Herston, QLD 4006, Australia; 2 UQ Centre for Clinical Research, The University of Queensland, Herston, QLD 4006, Australia

**Keywords:** respiratory tract infection, risk factor, sociodemographic factor, health disparity, age-standardized mortality rate, spatial analysis, time-trend analysis, South America

## Abstract

Lower respiratory infections (LRIs) are responsible for significant morbidity and mortality in Ecuador; however, evidence to support prevention strategies is limited. This study aimed to identify age-specific trends, spatial patterns, and sociodemographic risk factors of LRI mortality in Ecuador between 2012–2022, utilizing national mortality data sourced from the Ecuadorian National Institute for Statistics and Censuses (INEC). Age-sex-specific trend analysis was performed using Joinpoint regression. LRI age-standardized mortality rates (ASMRs) were mapped by province of death, and percentage change was calculated between 2012 and 2019. Multivariable logistic regression was performed to assess risk factors pre- and post-2020. A change in trend in LRI mortality rate, from a decreasing trend to a marginal increasing trend, was identified for both genders in children aged 0–4 and 5–15 years. There were significant increasing trends for males (2014–2019 APC: 2.21%, 95% CI: 0.57, 6.70) and females (2016–2019 APC: 4.62%, 95% CI: 0.84, 10.58) aged ≥ 70 years. From 2012 to 2019, the highest average LRI ASMR was in Guayas (30.90 deaths per 100,000 inhabitants), and the greatest percentage increase was observed in Orellana (419.54%). Before 2020, LRI mortality, compared to deaths of other causes, was significantly associated with sex, age, education, ethnicity, place of death and climate region, with major shifts post COVID-19 pandemic.

## 1. Introduction

Globally, lower respiratory infections (LRIs), defined as bronchiolitis or pneumonia, are responsible for significant morbidity, mortality and economic loss [1,2]. Despite a 41% decline in the global LRI mortality rate between 1990 and 2019, from 56.5 to 32.9 deaths per 100,000 population [1], LRI remained the leading infectious cause of death worldwide in 2019 [3]. In 2021, there were 344 million incident cases and 2.18 million deaths of LRI worldwide, excluding coronavirus disease (COVID-19) [1]. The burden of LRI mortality varies by age, geographic region, and sociodemographic indices, disproportionately affecting those aged <5 and >70 years, as well as those in low- and middle-income countries (LMIC) [1,4,5]. While the epidemiology is well studied in temperate, high-income countries, much less is known about LRI dynamics in tropical regions, in part due to complex seasonality and limited historical data [6,7,8].

LRI remains an important public health issue in Ecuador. Between 2011 and 2015, acute respiratory infections were estimated to account for an average loss of productivity of USD 152.16 million per year [9]. In 2021, LRI (excluding COVID-19) was the fifth leading cause of mortality [10], responsible for 337,000 incident cases and 4530 deaths [1]. Ecuador was severely impacted by the COVID-19 pandemic, with estimates indicating a 64% excess death rate, placing Ecuador among the highest-impacted countries globally, and highlighting the need for improved health emergency planning [11,12].

The leading risk factors of LRI mortality in Ecuador are largely modifiable, including exposure to ambient particulate matter pollution, low temperature and lack of access to handwashing facilities [13,14]. Such risk factors vary by geographic region and socio-economic status (SES) [15].

The burden of LRI is changing over time and varies widely by age, gender, region, and sociodemographic factors [16,17]. Therefore, maintaining an up-to-date understanding of LRI mortality dynamics is important for evaluating the impact on health and informing public health strategies. Limited studies have assessed temporal and spatial trends of LRI mortality in Ecuador in recent years, and previous studies have limited their focus to specific age groups (i.e., <5 and ≥50 years) [18,19,20], geographic areas [20], or etiologies [19,21]. Furthermore, recent studies investigating risk factors have largely focused on COVID-19 [22,23,24].

This study aimed to identify temporal trends in LRI mortality in Ecuador between 2012 and 2022. The specific objectives were to identify (1) age-specific trends, (2) spatial patterns, and (3) sociodemographic risk factors of LRI mortality.

## 2. Materials and Methods

### 2.1. Study Design and Setting

**Figure 1:**
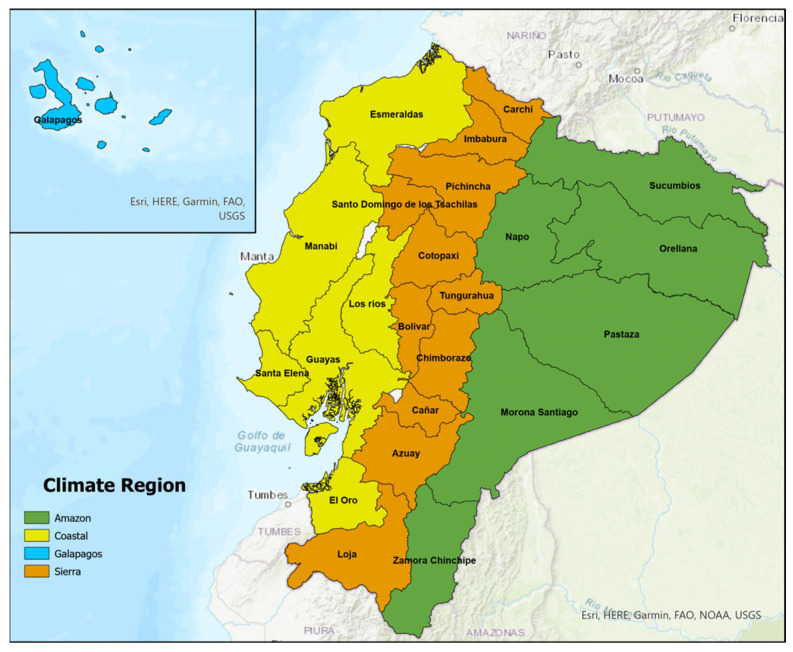
Map of Ecuador, separated by province and climate region. Map generated using ArcGIS Pro version 3.1.1 (Esri; Redlands, CA, USA).

This population-based cross-sectional study included all registered deaths in Ecuador from 1 January 2012 to 31 December 2022, inclusive. Ecuador encompasses 24 provinces, categorized into four climate regions of significant geographical, social, and economic differences: the Sierra (Andean Highlands), Amazon, Coastal, and Insular (Galapagos) regions [25] (Figure 1).

### 2.2. Data Sources

National mortality data were sourced from the Ecuadorian National Institute for Statistics and Censuses (INEC), which contains cause-of-death data and sociodemographic information [26]. Population estimates were sourced from the World Bank population projection database [27]. Age-specific province population data were sourced from INEC [28].

### 2.3. Outcome and Covariates

The outcome of interest was LRI-attributed mortality. Cause of death was assigned using the underlying cause of death, coded by the International Statistical Classification of Diseases and Related Health Problems, 10th revision (ICD-10). LRI was defined as bronchiolitis or pneumonia, using an established ICD-10 case definition as described by the Global Burden of Disease Study [1], and COVID-19 (Appendix A). Analyzing COVID-19 separately was considered due to the public health impact of the disease. However, due to a large peak in unspecified viral pneumonia mortality during 2020–2021, COVID-19 was grouped with LRI for the main analysis to comprehensively report trends and reduce risk of misclassification bias. Analyses were separated into pre-2020 and post-2020 periods.

Potential predictor variables were selected based on available data and associations reported in the literature [22,23,24,29,30,31]. Variables included level of education, area of residence, ethnicity, age, sex, place of death, and climate region of death. Variable classifications are provided in Appendix B.

### 2.4. Statistical Analyses

Descriptive statistics were calculated by cause of death and sociodemographic variables. Crude mortality rates were calculated per 100,000 inhabitantss as the number of deaths divided by the total population. Descriptive statistics, mortality rates and logistic regression were performed using STATA/SE version 18.0 (College Station, TX, USA; StataCorp LLC). Age-specific trend analysis was performed using Joinpoint Regression Analysis version 5.2 (Bethesda, MD, USA; Statistical Research and Applications Branch, National Cancer Institute). Spatial mapping was performed using ArcGIS Pro version 3.1.1 (Esri; Redlands, CA, USA). Missing data were excluded, except for place of death, which had a small percentage of missing data in the years 2020–2022. The percentage of missing data for each variable is provided in Table S1.

#### 2.4.1. Age-Specific Trends of LRI Mortality

Joinpoint Regression Analysis was performed to assess overall trends, whether there had been a statistically significant change in trend (joined by “joinpoints”), and whether the trend segments were statistically significant. The software starts at 0 joinpoints (straight line, indicating no change in trend) and tests whether adding more joinpoints is statistically significant, using Weighted Bayesian Information Criterion for model selection and the Empirical Quantile Method to calculate confidence intervals (CIs) [32]. Trend segment intervals are determined by Joinpoint analysis, and annual percentage change (APC) is calculated from the slope of the line to reflect the direction and magnitude of the trend for each trend segment. The average APC (AAPC) is computed as a weighted average of the APCs, with weights equal to the lengths of the APC segments [32].

Age-specific analysis was stratified by sex due to gender mortality differences [4]. Age-specific trend analysis was limited to 2012–2019, due to the large increase in mortality in 2020 that could not be accurately modeled using this method.

#### 2.4.2. Spatial Trends of LRI Mortality

Yearly age-standardized mortality rates (ASMRs) were mapped by province of death using ArcGIS Pro version 3.1.1. ASMR was estimated to adjust for differences in population age structure within and between provinces over time. ASMR was calculated per 100,000 inhabitants for each year, using the direct standardization method as described previously [33]. Briefly, for each year, ASMR was calculated by province as follows:

ASMR = Σ*r_i_ P_i_*/Σ*P_i_*

where *r_i_* is the age-specific mortality rate for age group *i* (5-year age groups, i.e., 0–4…80+ years), and *P_i_* is the standard population in age group *i* [34]. 2020 population estimates were used as the standard population.

Age-specific provincial population data were available only for the years 2012–2020. Average LRI ASMR was calculated between 2012 and 2019, and percentage change was calculated as $\frac{final\;value-initial\;value}{initial\;value}$ for 2012–2019 and 2019–2020.

#### 2.4.3. Risk Factors for LRI Mortality

Logistic regression was used to assess the association between sociodemographic variables and LRI mortality, compared to death from other causes. Univariable models were fitted to determine unadjusted associations between LRI mortality and each exposure variable in the overall data. Variables with significance *p* < 0.2 on univariate analysis were tested using multivariable logistic regression, and variables were sequentially removed to arrive at the most parsimonious model, in which variables with *p* < 0.05 were retained. The final multivariable model was run for the overall data, pre-COVID-19 pandemic (2013–2019), post-COVID-19 pandemic (2020–2022), and for each year. Separate analysis was performed for COVID-19 deaths. Age was grouped into 0–4, 5–59 and ≥70 years in accordance with high-burden age groups [1]. A mean variance inflation factor (VIF), with a threshold of 5.0, was used to assess multicollinearity among predictor variables using a linear approximation of the logistic model.

### 2.5. Ethical Approval

This study received ethics exemption (2024/HE000368) from UQ Human Research Ethics Committee.

## 3. Results

Between 2012 and 2022, there were 862,448 registered deaths in Ecuador, of which 89,697 (10.40%) were attributed to an LRI (Table 1). Most LRI-attributed deaths were male (58.96%), aged ≥70 years of age (55.57%), and Ecuadorian (“Mestizo”) ethnicity (82.15%). Most LRI deaths occurred in the coastal region (50.63%), in a public hospital (64.17%), in urban areas (81.50%), and were individuals with primary/basic education as the highest level obtained (46.99%). Descriptive statistics are also shown by etiology in Table 1. Descriptive statistics are presented separately for 2012–2019 and 2020–2022 (Tables S3 and S4).

**Figure 2:**
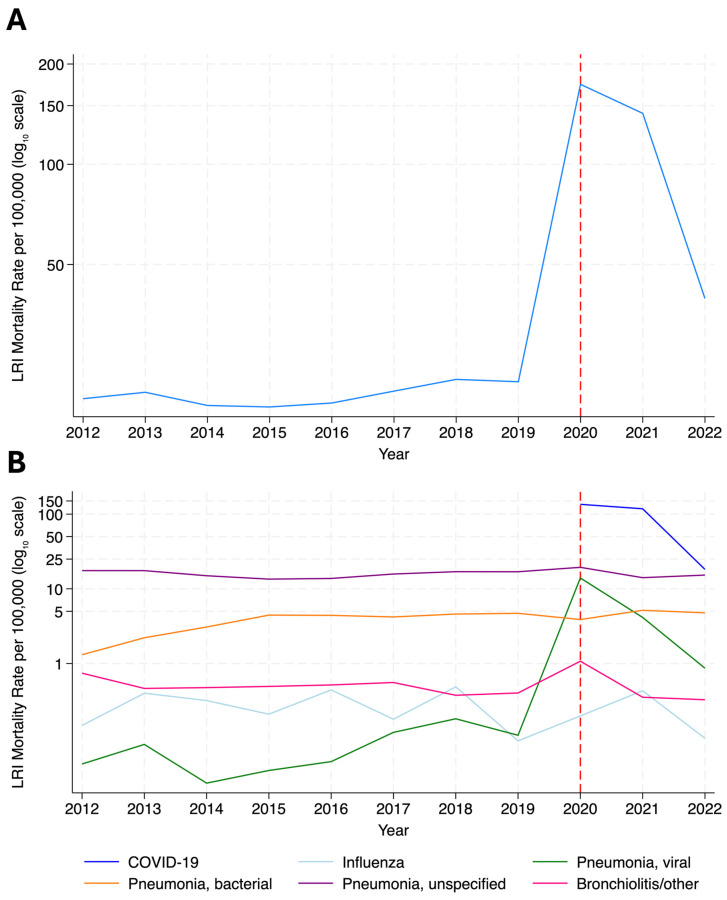
Lower respiratory infection (LRI) mortality rate (log_10_ scale) in Ecuador, 2012–2022. (**A**) Total LRI mortality rate per 100,000 inhabitants (**B**) LRI mortality rate per 100,000 inhabitants by high-burden etiologies. Y axis is presented on log_10_ scale for both (**A**,**B**). Rates per 100,000 inhabitants are calculated using World Bank population estimates. Dashed red line indicates year of COVID-19 outbreak, first detected in Ecuador on 29 February 2020. *N* = 89,697.

From 2012 to 2019, the crude LRI mortality rate increased from 19.78 to 22.24 deaths per 100,000 inhabitants, followed by a substantial increase in 2020 (173.79 deaths per 100,000), associated with COVID-19, before decreasing to 39.56 per 100,000 in 2022. The leading LRI-attributed causes of death were COVID-19 (*n* = 48,074, 53.60%) and pneumonia (unspecified (*n* = 27,589, 30.76%), unspecified bacterial (*n* = 6767, 7.54%) and unspecified viral (*n* = 3325, 3.71%)) (Figure 2; Table S2).

### 3.1. Age-Specific Trends of LRI Mortality

Due to the large increase in LRI mortality in 2020, trends could not be accurately modeled between 2012 and 2022 for older age groups (Table S5). Table 2 presents age-specific trends in LRI mortality between 2012 and 2019. On average, between 2012 and 2019, there was a 1.81% and 1.91% increase in LRI mortality rate per year for males and females of all ages, respectively.

Different patterns were seen by age group and gender. In the 0–4-year age group, there was an initial decreasing trend for males (2012–2014 Annual percentage change [APC]: −14.01%, 95% confidence interval [CI]: −20.19, −5.52) and females (2012–2015 APC: −9.90%, 95% CI: −18.51, −4.07), followed by a non-significant increasing trend in later years (males: 2014–2019 APC: 2.16%, 95% CI: −0.48, 8.24; females 2015–2019 APC: 2.29%, 95% CI: −2.47, 13.63). Similar patterns were observed in the 5–14-year age group. Trends remained stable for those aged 15–49 years. There was a 2.82% and 2.84% increase in the average mortality rate per year for males and females aged 50–69 years, respectively. For those aged ≥70 years, there was an initial decreasing trend for males (2012–2014 APC: −6.61, 95% CI: −10.49, −0.84) and females (2012–2016 APC: −2.10, 95% CI: −7.69, 0.78), followed by a significant increasing trend for both genders in later years (males: 2014–2019 APC: 2.21%, 95% CI: 0.57, 6.70; females: 2016–2019 APC: 4.62%, 95% CI: 0.84, 10.58) (Table 2).

### 3.2. Spatial Trends of LRI Mortality

**Figure 3:**
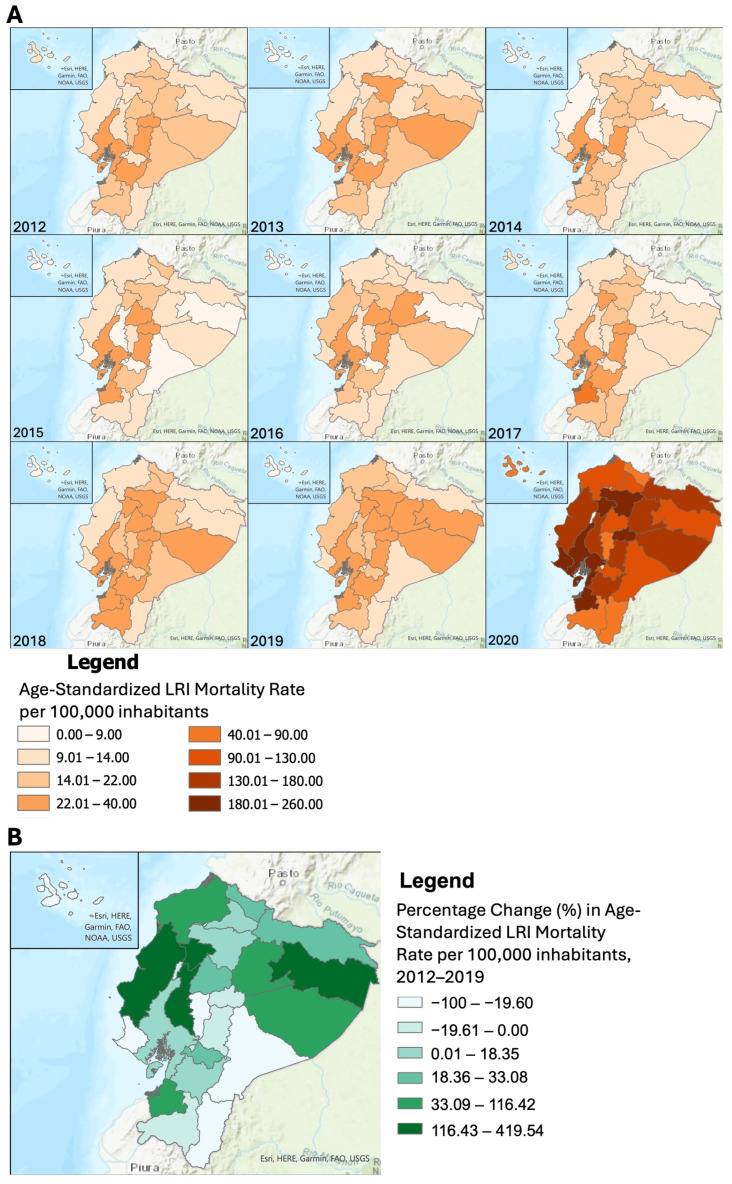
Spatial distribution of lower respiratory infection (LRI) age-standardized mortality rate (ASMR) per 100,000 inhabitants, Ecuador: (**A**) Yearly LRI ASMR per 100,000 inhabitants by province of death, 2012–2020. (**B**) Percent change (%) LRI ASMR per 100,000 inhabitants by province of death, 2012–2019.

Between 2012 and 2019, the average LRI ASMR was highest in Guayas (30.90 deaths per 100,000 inhabitants), followed by Chimborazo (28.87 deaths per 100,000 inhabitants), and the lowest in the Galapagos (3.91 deaths per 100,000 inhabitants) and undelimited zones (0.47 deaths per 100,000 inhabitants), with little change over time. Many provinces in the central Sierra region (Chimborazo, Tungurahua, Cotopaxi) showed high LRI ASMR, with increasing rates in many other provinces in the Sierra and Amazon regions in 2018 and 2019 (Figure 3, Table S6).

Between 2012 and 2019, the greatest percentage increase in LRI ASMR was observed in Orellana (419.54%) (Amazon region) from 6.59 deaths to 34.26 deaths per 100,000 inhabitants. This was followed by Santo Domingo de los Tsachilas (208.27%) (Sierra region), Manabi (148.23%), and Los Rios (123.73%) (Coastal region). Morona Santiago (Amazon region) showed the greatest percentage decrease (−57.32%) from 20.89 to 8.92 deaths per 100,000 from 2012 to 2019.

In 2020, the highest LRI ASMR were recorded in El Oro (Coastal region) (256.62 deaths per 100,000, respectively) and the lowest in Bolivar (Sierra region) (64.90 deaths per 100,000 inhabitants), with no deaths in undelimited zones (Figure 3, Table S6).

### 3.3. Risk Factors for LRI Mortality

Between 2013 and 2019, female sex (adjusted odds ratio [aOR]: 1.04, 95% CI: 1.01, 1.07), age groups 0–4 years (aOR: 1.61, 95% CI:1.48, 1.75) and ≥70 years (aOR: 2.54, 95% CI: 2.45, 2.63), indigenous ethnicity (aOR: 1.35, 95% CI: 1.26, 1.44) and death in a private hospital (aOR: 1.06, 95% CI: 1.02, 1.10) were associated with increased odds of LRI mortality, compared to death from other causes. “Other” ethnicities (aOR: 0.89, 95% CI: 0.84, 0.95), primary level education (aOR: 0.94, 95% CI: 0.90, 0.97), death at home (aOR: 0.39, 95% CI: 0.38, 0.41) or other establishment (aOR: 0.22, 95% CI: 0.20, 0.25) and the Amazon region (aOR: 0.66, 95% CI: 0.60, 0.73) were associated with reduced odds of LRI mortality, compared to death from other causes (Table 3). The odds of LRI mortality among individuals of indigenous ethnicity decreased from 2014 (aOR: 1.84, 95% CI: 1.45, 2.33) to 2019 (aOR: 1.35, 95% CI: 1.16, 1.57) compared to the Ecuadorian group (Tables S9–S14, Figure S5).

There were major shifts in risk factors in 2020–2022. Female sex (aOR: 0.77, 95% CI: 0.76, 0.79), rural residence (aOR: 0.88, 95% CI: 0.86, 0.90), indigenous ethnicity (aOR: 0.87, 95% CI: 0.82, 0.92), and death outside a public hospital were associated with reduced odds of LRI mortality, compared to death from other causes. Primary (aOR: 1.37, 95% CI: 1.33, 1.42) or secondary and above education (aOR: 1.22, 95% CI: 1.18, 1.27), those aged ≥70 years (aOR: 1.08, 95% CI: 1.05, 1.10), and the Sierra region (aOR: 1.22, 95% CI: 1.19, 1.24) were associated with increased odds of LRI mortality (Table 3). When restricting the analysis to COVID-19 deaths, the magnitude of the change in association increased for all variables except sex and area of residence (Table 4).

## 4. Discussion

This study elucidates recent temporal trends, spatial patterns and socio-demographic risk factors of LRI mortality in Ecuador. Overall, the crude LRI mortality rate increased prior to the COVID-19 pandemic and did not return to pre-pandemic rates in 2022.

There was uneven progress in reducing LRI mortality rates across different age groups prior to the COVID-19 pandemic. The significant reduction in LRI mortality rate for children aged 0–4 years between 2012 and 2015 is supported by previous studies [18,19], and coincides with the introduction of the pneumococcal conjugate vaccination (PCV) for infants in 2010 [35], as well as improvements in access to healthcare services [36]. However, the decreasing trend was not sustained, and marginal increases from 2014 and 2015 highlight the importance of continual monitoring of trends and vaccination uptake. In the context of national immunization coverage, PCV coverage peaked at 94% in 2012 and decreased to 83% in 2019 [35], with child immunization programs significantly impacted by the COVID-19 pandemic [37]. This study also identified similar trends in the 5–14 age group and stable trends in the 15–49 age group, which, to the best of our knowledge, had not yet been reported.

The findings in older adults extend from a study conducted by Sotomayor et al., which reported pneumonia mortality steadily increased in adults aged 50–64 and ≥65 years from 2005 to 2013, before slowing decreasing to 2015 [19]. This study shows that LRI mortality rate increased through 2019 for adults aged 50–69 years. Furthermore, in the age group with the highest burden, ≥70 years, there were decreasing trends, followed by significant increasing trends for both sexes towards the end of the study period.

In an aging population, efforts to reduce LRI mortality in older adults are particularly important to manage the burden on the healthcare system [9,38]. Further understanding of the change in trend and etiology in this age group is important to inform the efficacy of public health strategies.

When adjusting for differences in the population age structure, between 2012 and 2019, the average LRI mortality rate was highest in Guayas, followed by Chimborazo, with little change over time, indicating a need to identify public health strategies to mitigate the ongoing burden in these provinces. Furthermore, many provinces in the high-altitude central Sierra region had a high LRI ASMR, particularly in 2018–2019. Oritz-Prado et al. observed that pneumonia mortality increased at altitudes above 3500 m in children aged under 5 years, possibly due to reduced access to healthcare services [39].

This study suggests that Orellana, in the Amazon region, showed the greatest increase in LRI ASMR prior to the COVID-19 pandemic. Orellana is of particular concern due to the high percentage of poverty [40] and households without basic sanitation facilities [41], which is associated with poor health outcomes and vulnerability to LRI [42,43]. Furthermore, Orellana also had the lowest density of hospital beds in the country [44]. Gomez-Garcia et al. reported Orellana had the greatest increase in asthma-attributed morbidity rates between 2016 and 2019 [45], which is a known risk factor for community-acquired pneumonia [46], potentially due to environmental and occupational exposures to oil refineries in the province [45].

In this analysis, El Oro had the highest LRI ASMR during the COVID-19 pandemic in 2020. However, studies of excess death highlight substantial underreporting of COVID-19-attributed mortality, with Guayas and Santa Elena experiencing the highest excess death rates in 2020 [11,12,29].

The analysis of risk factors was separated into pre-pandemic and post-pandemic periods. Prior to the COVID-19 pandemic in 2020, indigenous ethnicity and age groups 0–4 years and ≥70 years were significant risk factors for LRI mortality, compared to death from other causes. There was a modest effect size for female sex and death in a private hospital, whereas “other” ethnicity, primary level education, death at home or other establishment and the Amazon region were associated with lower odds of LRI mortality.

Health inequalities in the indigenous population have been reported regardless of education, wealth, or area of residence [47]. Yearly analysis suggests improvements in ethnic disparities in LRI mortality between 2014 and 2019. The improvements may relate to a series of health reforms introduced between 2007 and 2017 [48], which reduced inequalities in access and utilization of health care services between 2006 and 2014 [36]. However, further progress is required to address inequities. Despite reported disparities in quality of healthcare for other ethnic minorities [49], there was evidence of decreased risk of LRI mortality for this subpopulation. However, this may reflect the classification of other ethnicities.

The analysis before the COVID-19 pandemic supports prioritizing interventions targeted to elderly adults and young children. Elderly adults have a higher risk of LRI mortality, in part due to age-related decline in the immune system and organ function, as well as the development of comorbidities [50]. Furthermore, while there were improvements in LRI mortality in children aged < 5 years, LRI remained the third leading cause of death in Ecuador for this age group in 2019, for which the leading risk factors are primarily preventable [13,14]. Interventions targeting the leading risk factors in older adults (ambient particulate matter, low temperature, lack of handwashing facilities and smoking), and children aged < 5 years (stunting, underweight, ambient particulate matter) may be beneficial [13,14].

Despite evidence of greater risk of pneumonia mortality in those with a lower level of education in Colombia [51], before 2020, there were only modestly reduced odds of LRI mortality, rather than death of different causes for adults with primary level education. Furthermore, while males have a higher mortality rate [4], females were more likely to die of an LRI rather than other causes.

People were less likely to die of LRI, rather than other causes, at home or in other establishments, compared to a public hospital in all years, which may indicate appropriate access to tertiary healthcare and healthcare-seeking behaviors when symptoms are severe. However, early intervention is important to prevent complications in severe cases [52]. Notably, increased risk of mortality in private hospitals has also been reported for other health conditions due to the substantial variation in facility quality [49].

The Amazon region experiences yearlong rainfall, high humidity and steady temperature, while the Coastal region experiences a tropical climate with distinct wet and dry seasons [53]. While climatic factors may influence respiratory virus transmission, studies have reported respiratory infections to be associated with the rainy season in the tropics [54,55,56]. The lower odds of LRI mortality in the Amazon region reported here may relate to social and demographic factors including lower population density [15], or underreporting due to limited surveillance and healthcare infrastructure [57].

Importantly, there were changes in the direction and magnitude of associations for many risk factors after the COVID-19 pandemic. These associations are in part supported by the literature, which reported increased risk of COVID-19 mortality for males [22,23,30], and those treated in a public health center [22,23]. However, some associations contrast with the literature, which suggests older age [22,23], low level of education [58], and the coastal region [30] as risk factors for the COVID-19 case fatality rate. There was conflicting evidence for the effect of living in rural areas [22,23], and unclear results for indigenous ethnicity [23,30,31]. There was substantial underreporting of COVID-19-attributed deaths in Ecuador during the COVID-19 pandemic [12,29]. The proportion of unreported deaths was higher in men, the coastal region, the elderly, and the indigenous population [11,12,29], which may explain the observed discrepancies. Additionally, this analysis is compares LRI mortality to deaths of other causes. Importantly, yearly analysis shows that the magnitude and direction of the associations trended towards pre-pandemic levels in 2022. However, as COVID-19 had significant societal and economic impacts in Ecuador [59], continued monitoring is of high importance.

This study has several limitations. Firstly, the analysis is dependent on the inherent quality and completeness of the mortality records. Peralta et al. found substantial disparities in the completeness and quality of the mortality registry by sex, age and province between 2001 and 2013, with underreporting in the Amazon region [57], which likely implicates the accuracy of our spatial and risk factor analysis if similar patterns exist in later years.

Secondly, there were limitations to the methods. To adjust for education as a proxy indicator of SES, and include all ages in the multivariable model, children aged < 20 years of age were categorized together. This may have reduced the magnitude of the association observed for the 0–4 year age group in the multivariable model, and results should be interpreted with caution.

Thirdly, this analysis does not consider vaccination status, population density, air pollution, co-comorbidities or smoking status, some of which are leading risk factors of LRI mortality in Ecuador [13,14]. It also does not consider interaction effects. Analysis of risk factors is compared to death from other causes and may not directly reflect risk in the overall healthy population although it provides important information to understand contributing factors related to LRI mortality.

This study also has key strengths. A large study size of all registered deaths in Ecuador enabled the comparison of trends in different regions and population groups, as well as the associations between multiple exposures in recent years.

## 5. Conclusions

LRI mortality varied by age, province and sociodemographic factors. The significant changes in trend for LRI mortality rate in high-burden age groups, 0–4 and ≥70 years, highlight the importance of ongoing surveillance to detect emerging trends to inform public health strategies. Spatial analysis identified consistently high LRI ASMR in Guayas and Chimborazo, and increasing rates in Orellana between 2012 and 2019. Prior to 2020, LRI mortality, compared to deaths from other causes, was significantly associated with sex, age, education, ethnicity, place of death and climate region, with inequities in the indigenous population. Importantly, there were major shifts in risk factors after the COVID-19 pandemic in 2020. These findings provide key information to guide further targeted studies and prioritization of resources to improve health outcomes related to LRI mortality in Ecuador.

## Figures and Tables

**Table 1:** Frequency of registered deaths in Ecuador, by cause of death and sociodemographic data, 2012–2022. Number (*N*) and column percents (%) are shown by lower respiratory infection (LRI) attributed death, LRI etiologies, and deaths from other causes.

Variable	Deaths from Other Causes *N* (%)	LRI (Total) *N* (%)	COVID-19 *N* (%)	Influenza *N* (%)	Pneumonia, Viral *N* (%)	Pneumonia, Bacterial *N* (%)	Pneumonia, Unspecified *N (*%)	Bronchiolitis/ Other *N (%)*	Total Deaths *N (%)*
Total	772,751 (89.60)	89,697 (10.40)	48,074 (5.57)	503 (0.06)	3459 (0.40)	7251 (0.84)	29,443 (3.41)	967 (0.11)	862,448 (100)
Sex									
Male	430,585 (55.72)	52,886 (58.96)	30,393 (63.22)	255 (50.70)	2253 (65.13)	3861 (53.25)	15,619 (53.05)	505 (52.22)	483,471 (56.06)
Female	342,166 (44.28)	36,811 (41.04)	17,681 (36.78)	248 (49.30)	1206 (34.87)	3390 (46.75)	13,824 (46.95)	462 (47.78)	378,977 (43.94)
Age Group									
0–4 years	25,553 (3.31)	2617 (2.92)	100 (0.21)	34 (6.76)	51 (1.47)	229 (3.16)	1999 (6.79)	204 (21.10)	28,170 (3.27)
5–14 years	19,930 (2.58)	1641 (1.83)	117 (0.24)	19 (3.78)	29 (0.84)	162 (2.23)	1228 (4.17)	86 (8.89)	21,571 (2.50)
15–49 years	143,089 (18.52)	8460 (9.43)	4737 (9.85)	88 (17.50)	303 (8.76)	589 (8.12)	2686 (9.12)	57 (5.89)	151,549 (17.57)
50–69 years	180,611 (23.37)	27,051 (30.16)	19,535 (40.64)	143 (28.43)	1357 (39.23)	1298 (17.90)	4585 (15.57)	133 (13.75)	207,662 (24.08)
≥70 years	403,140 (52.17)	49,842 (55.57)	23,522 (48.93)	219 (43.54)	1719 (49.70)	4972 (68.57)	18,923 (64.27)	487 (50.36)	452,982 (52.52)
Missing information	428 (0.06)	86 (0.10)	63 (0.13)	–	–	1 (0.01)	22 (0.07)	–	514 (0.06)
Area of Residence									
Urban	595,463 (77.06)	73,099 (81.50)	39,685 (82.55)	358 (71.17)	3251 (93.99)	5872 (80.98)	23,254 (78.98)	679 (70.22)	668,562 (77.52)
Rural	177,271 (22.94)	16,598 (18.50)	8389 (17.45)	145 (28.83)	208 (6.01)	1379 (19.02)	6189 (21.02)	288 (29.78)	193,869 (22.48)
Missing information	17 (<0.01)	–	–	–	–	–	–	–	17 (<0.01)
Level of Education									
No formal education	170,733 (22.09)	13,581 (15.14)	4506 (9.37)	102 (20.28)	290 (8.38)	1623 (22.38)	6831 (23.20)	229 (23.68)	184,314 (21.37)
Primary/Basic	311,974 (40.37)	42,145 (46.99)	25,969 (54.02)	184 (36.58)	1620 (46.83)	2914 (40.19)	11,202 (38.05)	256 (26.47)	354,119 (41.06)
Secondary and above	171,175 (22.15)	21,254 (23.70)	12,903 (26.84)	113 (22.47)	998 (28.85)	1696 (23.39)	5437 (18.47)	107 (11.07)	192,429 (22.31)
Child aged <20 years	59,406 (7.69)	4709 (5.25)	286 (0.59)	57 (11.33)	90 (2.60)	453 (6.25)	3526 (12.98)	297 (30.71)	64,115 (7.43)
Missing information	59,463 (7.69)	8008 (8.93)	4410 (9.17)	47 (9.34)	461 (13.33)	565 (7.79)	2447 (8.31)	78 (8.07)	67,471 (7.82)
Ethnicity									
Indigenous	37,074 (4.80)	2955 (3.29)	943 (1.96)	56 (11.13)	41 (1.19)	256 (3.53)	1561 (5.30)	98 (10.13)	40,029 (4.64)
Ecuadorian	585,775 (75.80)	73,684 (82.15)	41,944 (87.25)	380 (75.55)	2849 (82.36)	5919 (91.63)	21,985 (74.67)	607 (62.77)	659,459 (76.46)
Other	49,428 (6.40)	3404 (3.79)	1398 (2.91)	20 (3.98)	117 (3.38)	397 (5.48)	1385 (4.70)	87 (9.00)	52,832 (6.13)
Missing information	100,747 (13.00)	9654 (10.76)	3789 (7.88)	47 (9.34)	452 (13.07)	679 (9.36)	4512 (15.32)	175 (18.10)	110,128 (12.77)
Place of Death									
Public hospital	228,717 (29.60)	57,560 (64.17)	38,101 (79.25)	248 (49.30)	1469 (42.47)	3224 (44.46)	14,296 (48.55)	222 (23.96)	286,277 (33.19)
Private hospital	83,019 (10.74)	11,854 (13.22)	4443 (9.24)	53 (10.54)	517 (14.95)	1836 (25.32)	4937 (16.77)	68 (7.03)	94,873 (11.00)
Home	391,939 (50.72)	18,563 (20.70)	4882 (10.16)	186 (36.98)	1444 (41.75)	2060 (28.41)	9349 (31.75)	642 (66.39)	410,502 (47.60)
Other	67,344 (8.71)	1200 (1.34)	245 (0.51)	16 (3.18)	8 (0.23)	108 (1.49)	794 (2.70)	29 (3.00)	68,544 (7.95)
Missing information	1732 (0.22)	520 (0.58)	403 (0.84)	–	21 (0.61)	23 (0.32)	67 (0.23)	6 (0.62)	2252 (0.26)
Climate Region of Death									
Coastal	401,966 (52.02)	45,414 (50.63)	22,758 (47.34)	86 (17.10)	3079 (89.01)	4501 (62.07)	14,490 (49.21)	500 (51.71)	447,380 (51.87)
Sierra	343,165 (44.41)	41,929 (46.75)	23,883 (49.68)	404 (80.32)	363 (10.49)	2643 (36.45)	14,191 (48.20)	445 (46.02)	385,094 (44.65)
Amazon	27,059 (3.50)	2311 (2.58)	1403 (2.92)	13 (2.58)	16 (0.46)	101 (1.39)	756 (2.57)	22 (2.28)	29,370 (3.41)
Insular/Galapagos	468 (0.06)	42 (0.05)	30 (0.06)	–	1 (0.03)	6 (0.08)	5 (0.02)	–	510 (0.06)
Exterior/Undelimited zones	93 (0.01)	1 (<0.01)	–	–	–	–	1 (<0.01)	–	94 (0.01)

**Table 2:** Age-specific trends of lower respiratory infection (LRI) mortality in Ecuador, 2012–2019. Average annual percentage change (AAPC) is calculated for 2012–2019, and annual percentage change (APC) is calculated for each trend segment. The 95% confidence intervals (CIs) are shown. *N* = 317 are missing.

	AAPC (95% CI) ^1^	Trend 1 ^1,2^		Trend 2 ^1,2^	
Age Group	2012–2019	Years	APC (95% CI)	Years	APC (95% CI)
All ages					
Male	1.81 (−0.21, −4.38)	2012–2015	−2.41 (−11.05, 6.12)	2015–2019	5.10 (−2.28, 14.46)
Female	1.91 (0.52, 3.29)	2012–2016	−1.51 (−7.06, 1.17)	2016–2019	6.65 (2.83, 12.94)
0–4 years					
Male	−2.75 (−4.57, −0.52)	2012–2014	−14.01 (−20.19, −5.52)	2014–2019	2.16 (−0.48, 8.24)
Female	−3.12 (−5.37, −0.72)	2012–2015	−9.90 (−18.51, −4.07)	2015–2019	2.29 (−2.47, 13.63)
5–14 years					
Male	−2.93 (−5.56, 0.46)	2012–2014	−12.07 (−20.84, −0.90)	2014–2019	1.05 (−5.04, 13.63)
Female	−2.42 (−7.93, 1.69)	2012–2017	−11.71 (−26.66, −5.69)	2017–2019	25.30 (−0.37, 50.82)
15–49 years					
Male	0.94 (−3.01, 5.18)	–	–	–	–
Female	−1.71 (−12.61, 10.89)	–	–	–	–
50–69 years					
Male	2.82 (−4.64, 11.97)	–	–	–	–
Female	2.84 (−0.12, 6.18)	–	–	–	–
≥70 years					
Male	−0.39 (−1.40, 1.16)	2012–2014	−6.61 (−10.49, −0.84)	2014–2019	2.21 (0.57, 6.70)
Female	0.72 (−0.68, 2.11)	2012–2016	−2.10 (−7.69, 0.78)	2016–2019	4.62 (0.84, 10.58)

^1^ Estimated by Joinpoint Regression model. ^2^ Trend segments are determined by Joinpoints (time points of change in trend) identified independently for each subgroup.

**Table 3:** Association between lower respiratory infection (LRI) mortality, compared to deaths from other causes, and sociodemographic risk factors in Ecuador. Multivariable model includes sex, age group, area of residence, ethnicity, level of education, place of death, and climate region of death. Mean variance inflation factor (vif) score 2013–2022 model: 1.34, 2013–2019 model: 1.32, 2020–2022 model: 1.38). Adjusted odds ratio (aOR) and 95% confidence intervals (CI) are shown.

	LRI-Attributed Death aOR (95% CI)		
Variable	2013–2022	2013–2019	2020–2022
Sex			
Male	Reference		
Female	0.85 (0.84, 0.87)	1.04 (1.01, 1.07)	0.77 (0.76, 0.79)
Age Group (years)			
0–4	1.37 (1.28, 1.47)	1.61 (1.48, 1.75)	1.03 (0.91, 1.17)
5–69	Reference		
≥70	1.32 (1.30, 1.35)	2.54 (2.45, 2.63)	1.08 (1.05, 1.10)
Area of Residence			
Urban	Reference		
Rural	0.97 (0.95, 0.99)	1.00 (0.96, 1.04)	0.88 (0.86, 0.90)
Ethnicity			
Ecuadorian	Reference		
Indigenous	1.07 (1.02, 1.11)	1.35 (1.26, 1.44)	0.87 (0.82, 0.92)
Other	0.65 (0.63, 0.68)	0.89 (0.84, 0.95)	0.62 (0.59, 0.66)
Level of Education			
No formal education	Reference		
Primary/Basic	1.57 (1.54, 1.61)	0.94 (0.90, 0.97)	1.37 (1.33, 1.42)
Secondary and above	1.50 (1.47, 1.55)	0.99 (0.94, 1.03)	1.22 (1.18, 1.27)
Child aged <20 years	0.73 (0.69, 0.77)	2.03 (1.88, 2.18)	0.27 (0.25, 0.30)
Place of Death			
Public hospital	Reference		
Private hospital	0.56 (0.54, 0.57)	1.06 (1.02, 1.10)	0.41 (0.40, 0.43)
Home	0.19 (0.18, 0.19)	0.39 (0.38, 0.41)	0.11 (0.11, 0.12)
Other/No information	0.06 (0.06, 0.07)	0.22 (0.20, 0.25)	0.03 (0.03, 0.03)
Climate Region of Death			
Coastal and Insular	Reference		
Sierra	1.12 (1.10, 1.13)	1.02 (0.99, 1.05)	1.22 (1.19, 1.24)
Amazon	0.95 (0.90, 0.99)	0.66 (0.60, 0.73)	0.97 (0.91, 1.03)

Data from 2012 were excluded due to missing data. Missing data were excluded from the multivariable model except for Place of Death, which had a small percentage of missing data in 2020–2022. 2013–2022 model: data are missing *N* (%) for Age Group 514 (0.06), Ethnicity 46,617 (5.83) and Level of Education 63,468 (7.94). 2013–2019 model: data are missing *N* (%) for Age Group 317 (0.06), Ethnicity 28,161 (5.84) and Level of Education 43,765 (9.07). 2020–2022 model: data are missing *N* (%) for Age Group 197 (0.06), Ethnicity 18,456 (5.83) and Level of Education 19,703 (6.22). For the Climate Region of Death, Insular (Galapagos) 510 (0.06) and Exterior/Undelimited zones 94 (0.01) were combined with Coastal 447,380 (51.87) due to limited data.

**Table 4:** The association between coronavirus disease (COVID-19) mortality, compared to deaths from other causes, and sociodemographic risk factors in Ecuador, 2013–2022. Multivariable model includes sex, age group, ethnicity, level of education, place of death, and climate region of death (mean variance inflation factor (vif) score: 1.38). Adjusted odds ratio (aOR) and 95% confidence intervals (CIs) are shown.

	COVID-19-Attributed Death
Variable	aOR (95% CI)
Sex	
Male	Reference
Female	0.77 (0.75, 0.78)
Age Group (years)	
0–4	0.56 (0.44, 0.72)
5–69	Reference
≥70	0.96 (0.94, 0.98)
Area of Residence	
Urban	Reference
Rural	NA
Ethnicity	
Ecuadorian	Reference
Indigenous	0.73 (0.68, 0.79)
Other	0.51 (0.48, 0.54)
Level of Education	
No formal education	Reference
Primary/Basic	2.56 (2.50, 2.67)
Secondary and above	2.39 (2.31, 2.48)
Child aged <20 years	0.16 (0.14, 0.18)
Place of Death	
Public hospital	Reference
Private hospital	0.34 (0.32, 0.35)
Home	0.09 (0.09, 0.09)
Other/No information	0.02 (0.02, 0.02)
Climate Region of Death	
Coastal and Insular	Reference
Sierra	1.40 (1.37, 1.44)
Amazon	1.38 (1.30, 1.48)

Data from 2012 were excluded due to missing data. Missing data were excluded from the multivariable model except for Place of Death, which had a small percentage of missing data in 2020–2022. Data are missing *N* (%) for Age Group 514 (0.06), Ethnicity 46,617 (5.83) and Level of Education 63,468 (7.94). For the Climate Region of Death, Insular (Galapagos) 42 (0.05) and Exterior/Undelimited zones 1 (<0.01) were combined with Coastal 45,414 (50.63) due to limited data. Area of Residence was not significant at *p* < 0.05 and was not included in the model.

## Data Availability

The datasets analyzed during the current study are available in the Ecuadorian National Institute for Statistics and Censuses (INEC) and World Bank Databank (population estimates and projections). INEC mortality datasets, by year: https://aplicaciones3.ecuadorencifras.gob.ec/BIINEC-war/index.xhtml?oe=DEFUNCIONES%20GENERALES (accessed on 13 December 2025); INEC population projection and estimate 2010–2020 dataset (Age projection Provinces 2010–2020 and national): https://www.ecuadorencifras.gob.ec/proyecciones-poblacionales-2010-2020/ (accessed on 20 February 2024); and World Bank population dataset: https://databank.worldbank.org/reports.aspx?source=2&series=SP.POP.TOTL&country=ECU (accessed on 20 February 2024).

## Supplementary Materials

The following supporting information can be downloaded at https://www.mdpi.com/article/10.3390/tropicalmed11010021/s1, Figure S1: Age-specific lower respiratory infection (LRI) mortality rate trend analysis 2012–2019 in Ecuador; Figure S2: Yearly association of lower respiratory infection (LRI) mortality and sex in Ecuador, 2012–2022; Figure S3: Yearly association of lower respiratory infection (LRI) mortality and age group (years) in Ecuador, 2012–2022; Figure S4: Yearly association of lower respiratory infection (LRI) mortality and area of residence in Ecuador, 2012–2022; Figure S5: Yearly association of lower respiratory infection (LRI) mortality and ethnicity in Ecuador, 2013–2022; Figure S6: Yearly association of lower respiratory infection (LRI) mortality and level of education in Ecuador, 2012–2022; Figure S7: Yearly association of lower respiratory infection (LRI) mortality and place of death in Ecuador, 2012–2022; Figure S8: Yearly association of lower respiratory infection (LRI) mortality and climate region of death in Ecuador, 2012–2022. Table S1: Descriptive statistics of mortality dataset by year, using expanded categories; Table S2: Number of lower respiratory infection (LRI) deaths in Ecuador by International Statistical Classification of Diseases and Related Health Problems, 10th Revision (ICD-10) code and year, 2012–2022; Table S3: Frequency of registered deaths in Ecuador, 2012–2019, by cause of death and socio-demographic data; Table S4: Frequency of registered deaths in Ecuador, 2020–2022, by cause of death and socio-demographic data; Table S5: Age-specific lower respiratory infection (LRI) mortality trend analysis, 2012–2022; Table S6: Lower respiratory infection (LRI) age-standardized mortality rates (ASMR) per 100,000 population per province, 2012–2020; Table S7: Association of sociodemographic risk factors and lower respiratory infection (LRI) mortality in Ecuador, 2012; Table S8: Association of sociodemographic risk factors and lower respiratory infection (LRI) mortality in Ecuador, 2013; Table S9: Association of sociodemographic risk factors and lower respiratory infection (LRI) mortality in Ecuador, 2014; Table S10: Association of sociodemographic risk factors and lower respiratory infection (LRI) mortality in Ecuador, 2015; Table S11: Association of sociodemographic risk factors and lower respiratory infection (LRI) mortality in Ecuador, 2016; Table S12: Association of sociodemographic risk factors and lower respiratory infection (LRI) mortality in Ecuador, 2017; Table S13: Association of sociodemographic risk factors and lower respiratory infection (LRI) mortality in Ecuador, 2018; Table S14: Association of sociodemographic risk factors and lower respiratory infection (LRI) mortality in Ecuador, 2019; Table S15: Association of sociodemographic risk factors and lower respiratory infection (LRI) mortality in Ecuador, 2020; Table S16: Association of sociodemographic risk factors and lower respiratory infection (LRI) mortality in Ecuador, 2021; and Table S17: Association of sociodemographic risk factors and lower respiratory infection (LRI) mortality in Ecuador, 2022.

## Abbreviations

The following abbreviations are used in this manuscript:

LRI	Lower Respiratory Infection
INEC	Ecuadorian National Institute for Statistics and Censuses
COVID-19	Coronavirus Disease
LMICs	Low- and Middle-Income Countries
SES	Socioeconomic Status
ICD-10	International Statistical Classification of Diseases and Related Health Problems, 10th Revision
ASMR	Age-Standardized Mortality Rate
AAPC	Average Annual Percentage Change
APC	Annual Percentage Change
CI	Confidence Interval
Vif	Variance Inflation Factor
OR	Odds Ratio
aOR	Adjusted Odds Ratio
PCV	Pneumococcal Conjugate Vaccination

## Appendix A

### Appendix A.1. LRI Case Definition

Lower respiratory infection (LRI) was defined as pneumonia or bronchiolitis using ICD-10 diagnostic criteria (A48.1, A70, B96.0–96.1, B97.21, B97.4–B97.6, J09–J11.89, J12–J13.9, J14–J14.0, J15–J15.8, J20–J21.9, J85.1, J91.0, P23.0–P23.4, U04–U04.9), as well as the following garbage codes (J15.9, J16–J19.6, J22–J22.9, P23, P23.5–P23.9) as per the Global Burden of Disease study [1]. Coronavirus disease 2019 (COVID-19) was also included, which is exclusively coded in the datasets as “COVID-19”. This was performed to reduce the risk of misclassification bias in this dataset, due to the large number of LRI deaths of unspecified origin, particularly during 2020–2021.

### Appendix A.2. ICD-10 Classification of LRI by High-Burden Etiologies

COVID-19 coded as “COVID-19”.

Influenza: J08, J09, J10.0, J10-1, J10.8, J10.10, J11.1, J11.8.

Pneumonia, viral: J12.0- J12.3, J12.8, J12.9, P23.0.

Pneumonia, bacterial: J13, J14, J15.0- J15.9, P23.1–P2.36.

Pneumonia, unspecified: J16.8, J18.1, J18.2, J18.8, J18.9, J85.1, P23.8, P23.9.

Bronchiolitis/Other: J219, J20.5, J20.8, J20.9, J21.8, J22.

## Appendix B

### Classification of Predictor Variables

Level of Education: (i) No formal education (“none”, “literacy center”, “infant garden”), (ii) Primary/Basic (“primary”, “basic education”), (iii) Secondary and above (“secondary education”, “baccalaureate”, “superior non-university”, “higher university”, “postgraduate”) and (iv) Child aged < 20 years.

As data for maternal education was not available, the level of education in adults (aged > 20 years) was used to minimize effects of collinearity with age in the multivariable model.

Climate Region of Death: (i) Costal region (Esmeraldas, Manabí, Los Ríos, Guayas, Santa Elena, El Oro), (ii) Sierra region (Carchi, Imbabura, Pichincha, Santo Domingo de los Tsáchilas, Cotopaxi, Tungurahua, Bolívar, Chimborazo, Cañar, Azuay, Loja), (iii) Amazonia region (Sucumbíos, Napo, Orellana, Pastaza, Morona Santiago, Zamora Chinchipe), (iv) Insular/Galapagos region (Galapagos islands), and (v) Exterior/Undelimited zones.

Ethnicity: (i) Indigenous (“Indigenous”), (ii) Ecuadorian (“Mestizo”), and (iii) Other (“Afro-Ecuadorian”, “Black”, “Mulato”, “Montubio”, “White”, “Other”).

In this dataset, “Indigenous” (4.64%) and “Ecuadorian” (74.46%) were the predominant self-reported ethnicities. Other ethnicities were grouped together due to insufficient observations for each year.

Place of Death: (i) Public hospital (“Establishments of the Ministry of Public Health”, “IESS establishments”, “other public establishment”), (ii) Private hospital (“Establishments of the Charity Board”, “Hospital Clinic/Private office”), (iii) Home (“home”) or (iv) Other/missing information. To retain data in the multivariable model, missing information was grouped with “other”, as missing data only occurred in 2020–2022, during the COVID-19 pandemic.

Age: Groupings were (i) 0–4, (ii) 5–14, (iii) 15–49, (iv) 50–69 and (v) ≥70 years for trend analysis as reported previously [1] and (i) 0–4, (ii) 5-69 and (iii) ≥70 years for anlysis of risk factors.
