# Supplementary material for: Temporal Trends in Lower Respiratory Infection Mortality in Ecuador, 2012–2022
Source: Trop Med Infect Dis. 2026 Jan 12;11(1):21. doi: 10.3390/tropicalmed11010021 (PMC12846537; doi:10.3390/tropicalmed11010021)
Supplement: Supplementary file 1 [file tropicalmed-11-00021-s001.zip › Supplementary_tropicalmed-4071531.pdf]

Supplementary Material

Supplement to: Temporal Trends in Lower Respiratory Infection Mortality in Ecuador, 2012–2022

Reena Krishna, Luis Furuya-Kanamori and Harriet Lawford

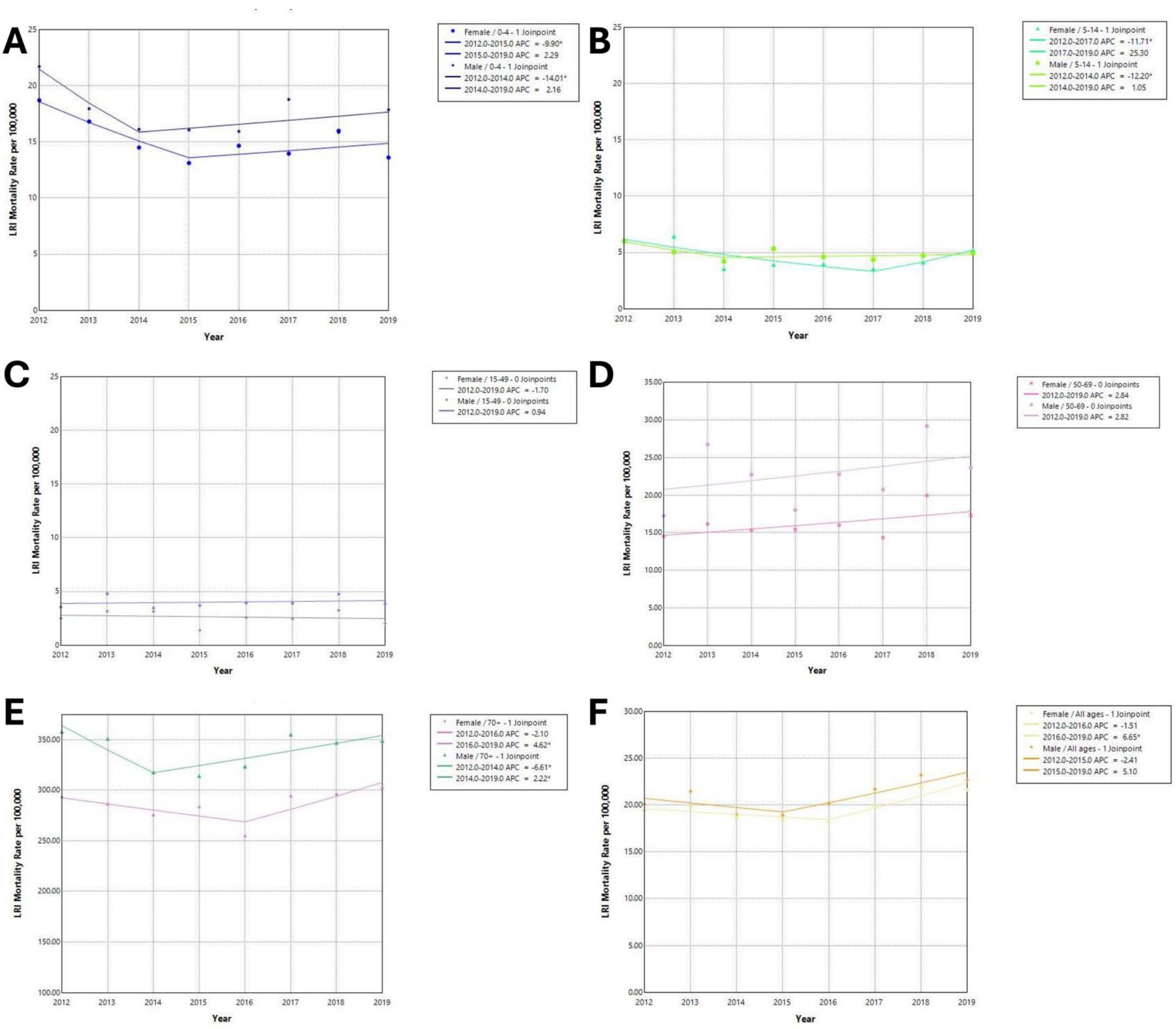

Figure S1: Age-specific lower respiratory infection (LRI) mortality rate trend analysis 2012–2019 in Ecuador. Data are shown stratified by sex for age categories (**A**) 0–4 years (**B**) 5–14 years (**C**) 15–49 years (**D**) 50–69 years (**E**) ≥70 years (**F**) all ages. Annual percentage change (APC) is shown for each trend segment. * Indicates statistically significantly different from 0 at the *p*=0.05 level. Age-groups are presented separately due to scale; y-axis scales are not the same. *N*=514 are missing from age-specific analysis.

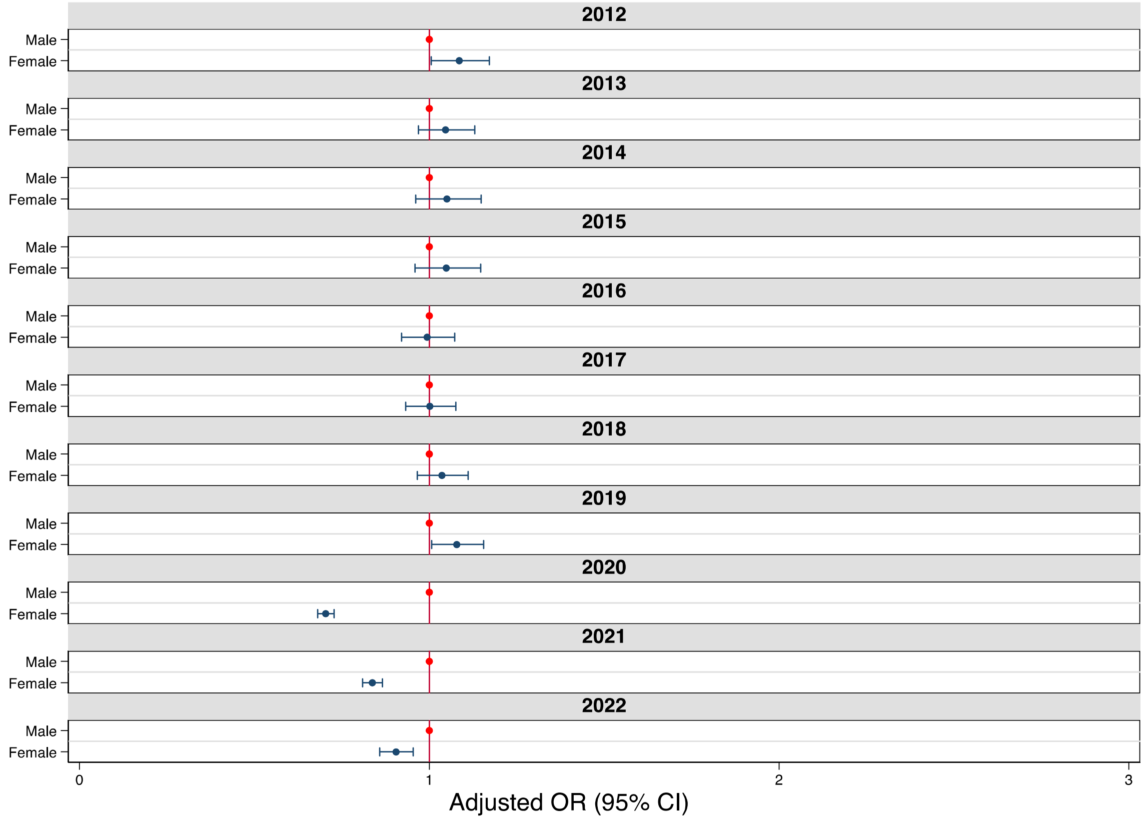

Figure S2: Yearly association of lower respiratory infection (LRI) mortality and sex in Ecuador, 2012–2022. Adjusted odds ratio (aOR) and 95% confidence intervals (CI) are shown for each year between 2012–2022. Multivariable model includes sex, age group, area of residence, level of education, ethnicity, place of death and climate region of death. Ethnicity was excluded from the multivariable model for 2012, due to no data. Associations with LRI mortality are compared with those for deaths from other causes.

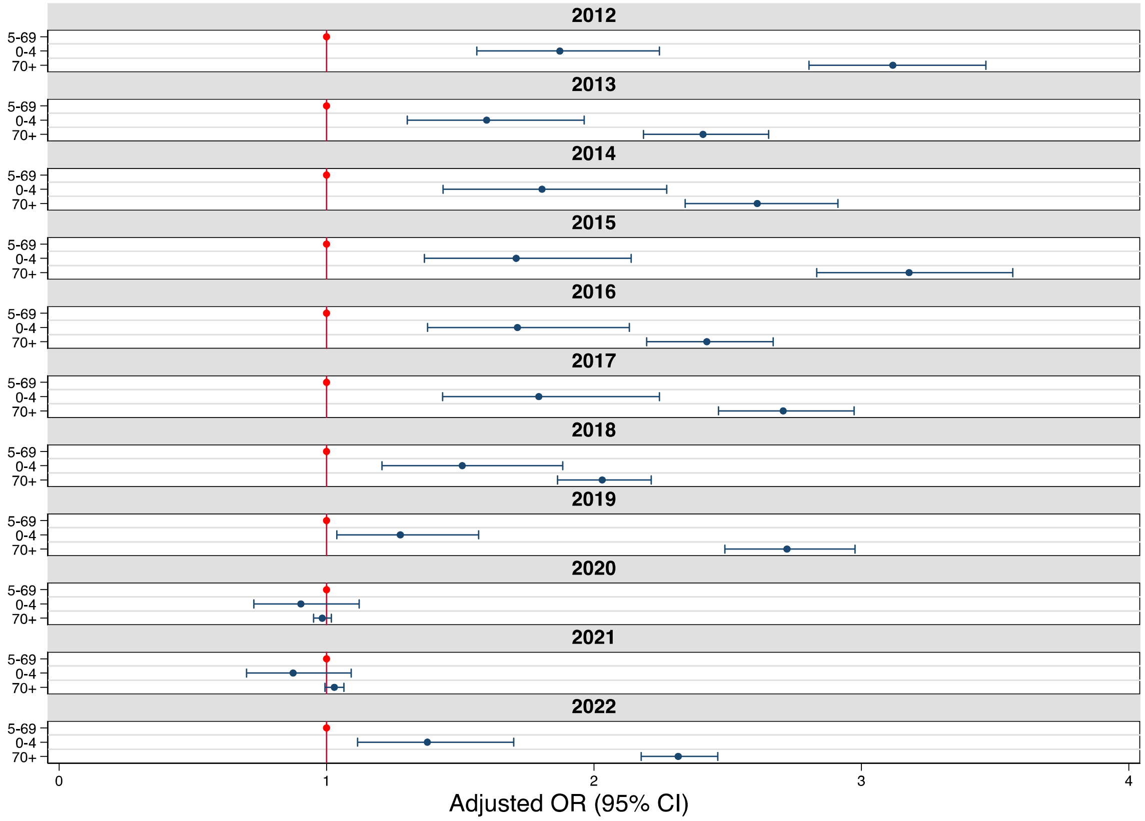

Figure S3: Yearly association of lower respiratory infection (LRI) mortality and age group (years) in Ecuador, 2012–2022. Adjusted odds ratio (aOR) and 95% confidence intervals (CI) are shown for each year between 2012–2022. Multivariable model includes sex, age group, area of residence, ethnicity, level of education, place of death and climate region of death. Ethnicity was excluded from the multivariable model for 2012, due to no data. Associations with LRI mortality are compared with those for deaths from other causes.

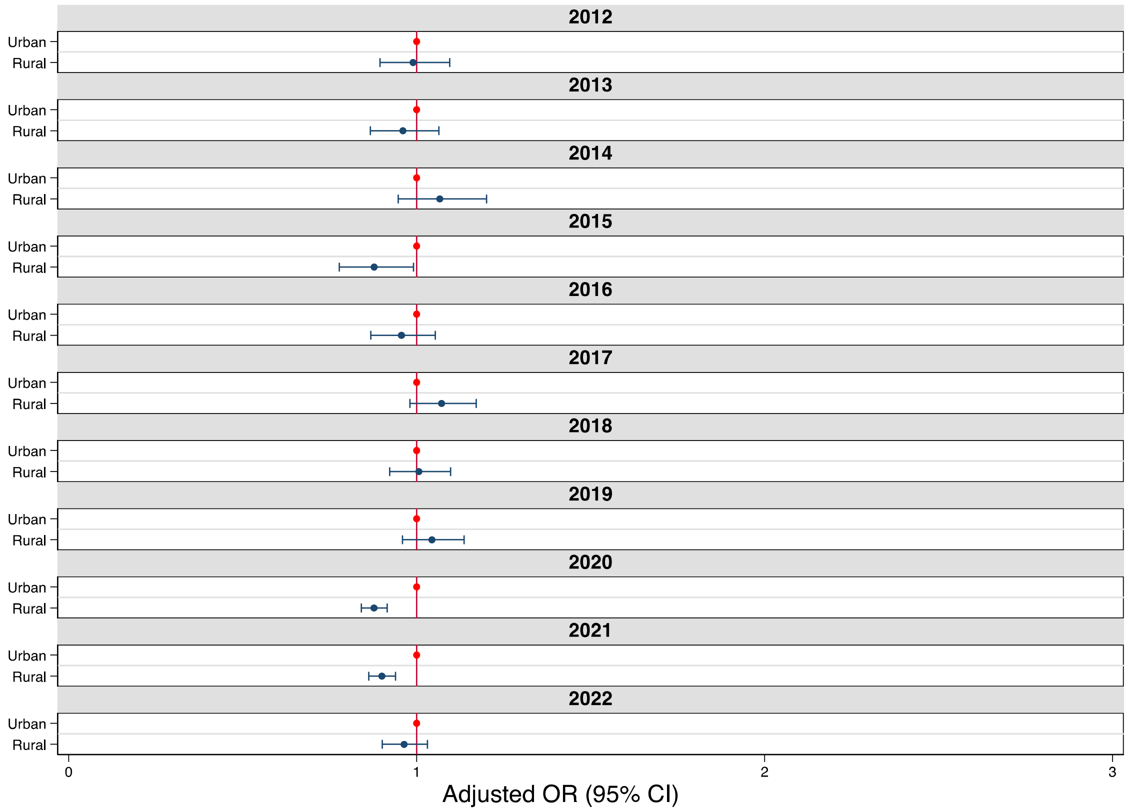

Figure S4: Yearly association of lower respiratory infection (LRI) mortality and area of residence in Ecuador, 2012–2022. Adjusted odds ratio (aOR) and 95% confidence intervals (CI) are shown for each year between 2012–2022. Multivariable model includes sex, age group, area of residence, ethnicity, level of education, place of death and climate region of death. Ethnicity was excluded from the multivariable model for 2012, due to no data. Associations with LRI mortality are compared with those for deaths from other causes.

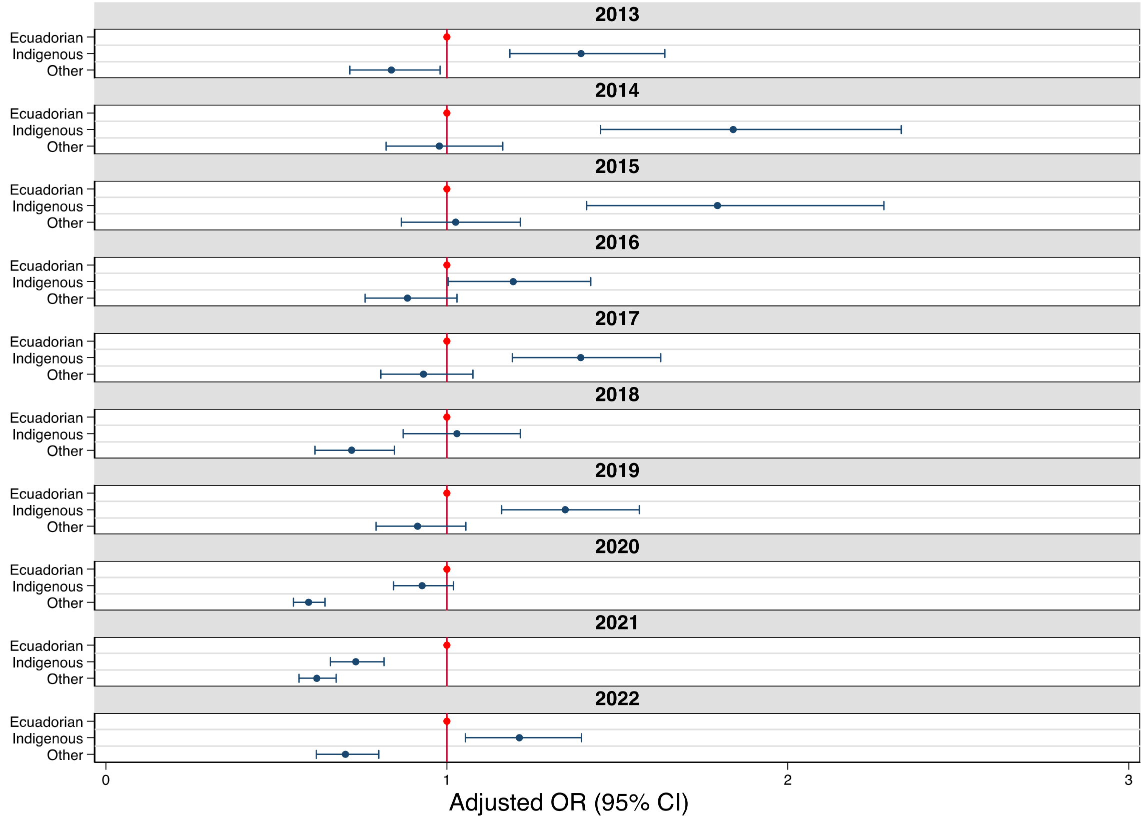

Figure S5: Yearly association of lower respiratory infection (LRI) mortality and ethnicity in Ecuador, 2013–2022. Adjusted odds ratio (aOR) and 95% confidence intervals (CI) are shown for each year between 2013–2022. Multivariable model includes sex, age group, area of residence, ethnicity, level of level of education, place of death and climate region of death. 2012 is not shown, due to no data. Associations with LRI mortality are compared with those for deaths from other causes.

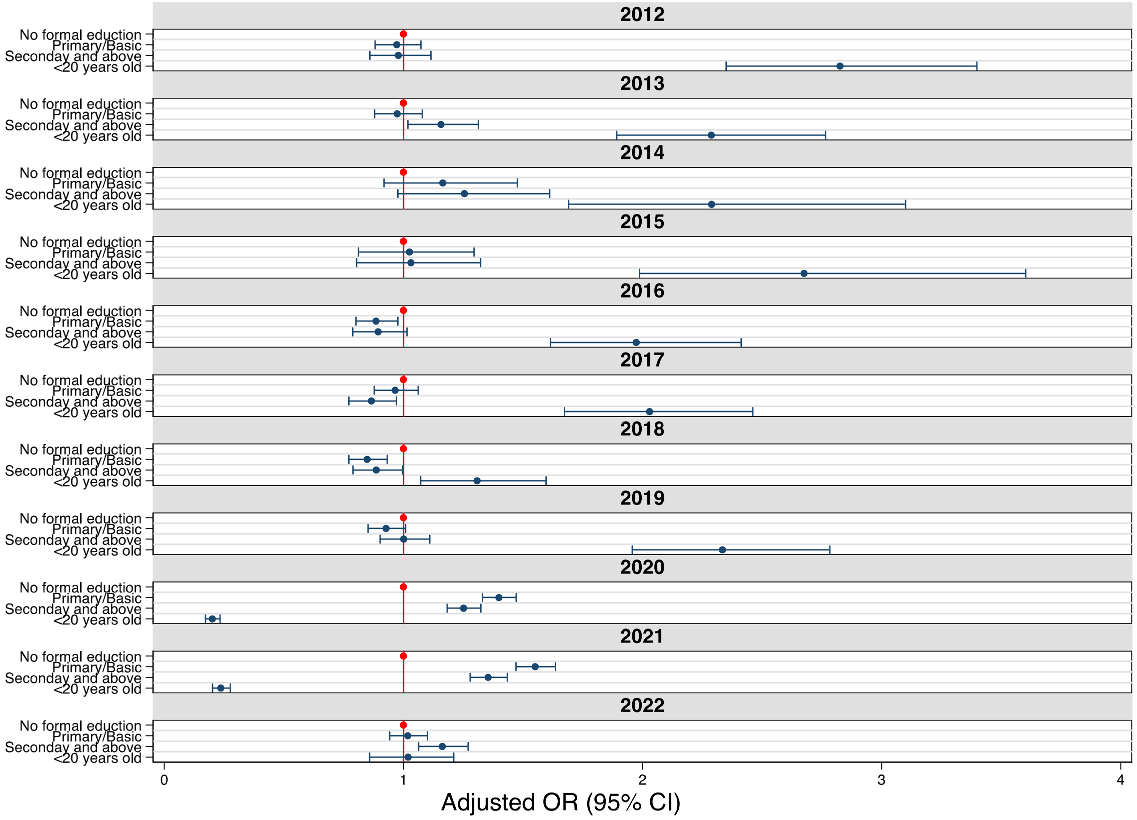

Figure S6: Yearly association of lower respiratory infection (LRI) mortality and level of education in Ecuador, 2012–2022. Adjusted odds ratio (aOR) and 95% confidence intervals (CI) are shown for each year between 2012–2022. Multivariable model includes sex, age group, area of residence, ethnicity, level of education, place of death and climate region of death. Ethnicity was excluded from the multivariable model for 2012, due to no data. Associations with LRI mortality are compared with those for deaths from other causes.

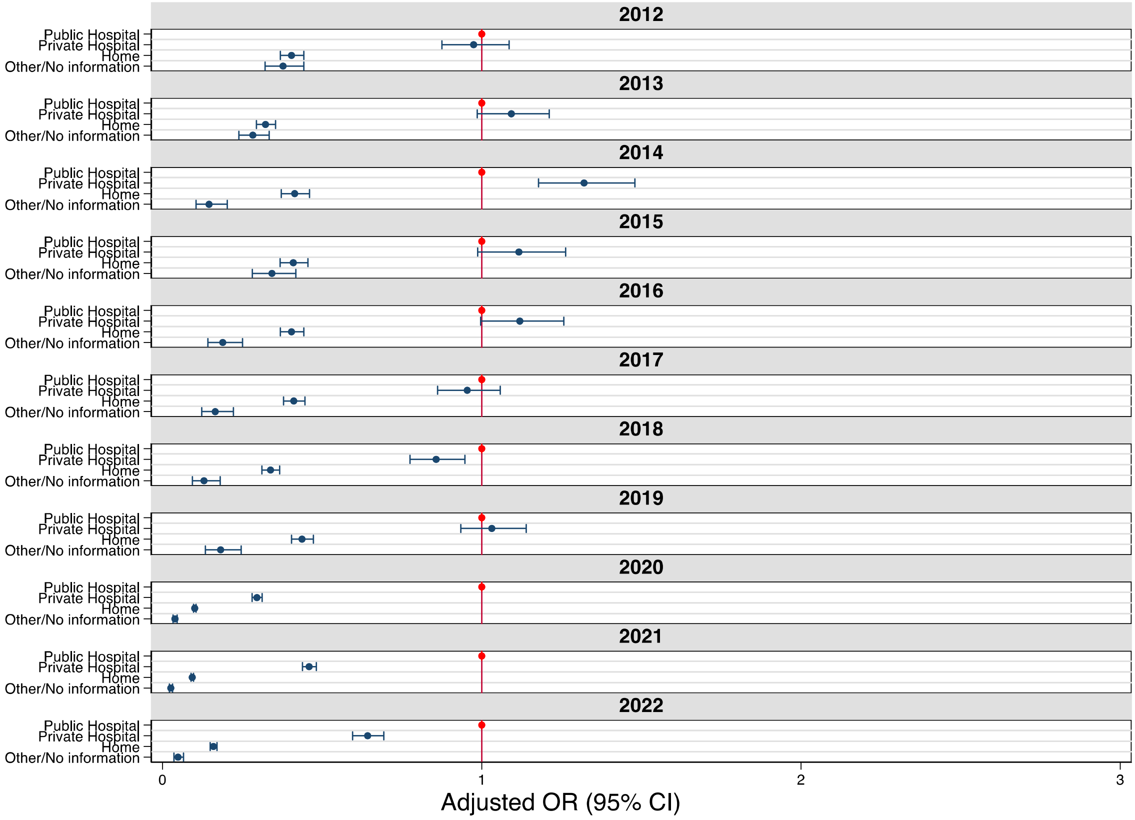

Figure S7: Yearly association of lower respiratory infection (LRI) mortality and place of death in Ecuador, 2012–2022. Adjusted odds ratio (aOR) and 95% confidence intervals (CI) are shown are each year between 2012–2022. Multivariable model includes sex, age group, area of residence, ethnicity, level of education, place of death and climate region of death. Ethnicity was excluded from the multivariable model for 2012, due to no data. Associations with LRI mortality are compared with those for deaths from other causes.

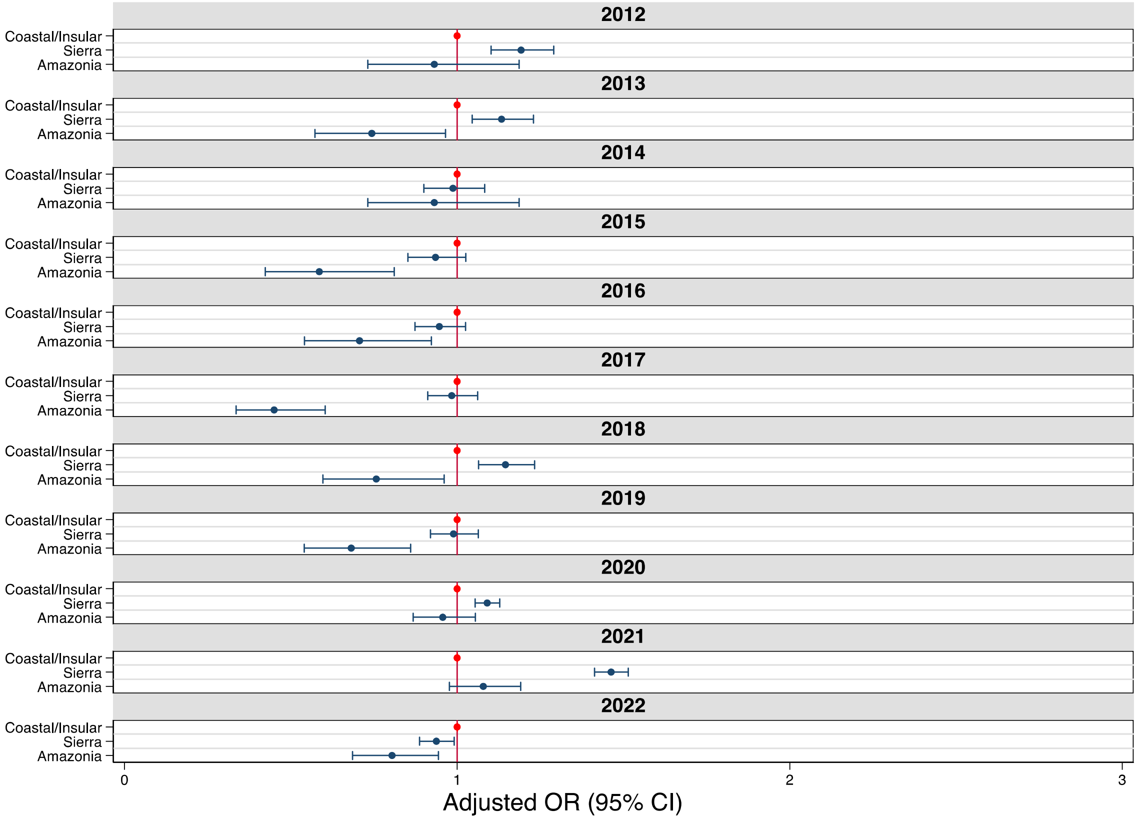

Figure S8: Yearly association of lower respiratory infection (LRI) mortality and climate region of death in Ecuador, 2012–2022. Adjusted odds ratio (aOR) and 95% confidence intervals (CI) are shown for each year between 2012–2022. Multivariable model includes sex, age group, area of residence, ethnicity, level of education, place of death and climate region of death. Ethnicity was excluded from the multivariable model for 2012, due to no data. Associations with LRI mortality are compared with those for deaths from other causes.

Table S1: Descriptive statistics of mortality dataset by year, using expanded categories. Number (*N*) and column percents (%) are shown. LRI = Lower respiratory infection.

Variable	2012 *N* (%)	2013 *N* (%)	2014 *N* (%)	2015 *N* (%)	2016 *N* (%)	2017 *N* (%)	2018 *N* (%)	2019 *N* (%)	2020 *N* (%)	2021 *N* (%)	2022 *N* (%)	Total *N* (%)
Total Deaths	63,511 (7.4)	63,104 (7.3)	64,770 (7.5)	66,598 (7.7)	68,848 (8.0)	70,841 (8.2)	72,789 (8.4)	75,355 (8.7)	117,030 (13.6)	107,648 (12.5)	91,954 (10.7)	862,448 (100)
Cause of Death												
Deaths from other causes	60,448 (95.2)	59,852 (94.8)	61,757 (95.3)	63,572 (95.5)	65,691 (95.4)	67,360 (95.1)	68,942 (94.7)	71,497 (94.9)	86,462 (73.9)	82,337 (76.5)	84,833 (92.3)	772,751 (89.6)
LRI mortality	3063 (4.8)	3252 (5.2)	3013 (4.7)	3026 (4.5)	3157 (4.6)	3481 (4.9)	3847 (5.3)	3858 (5.1)	30,568 (26.1)	25,311 (23.5)	7121 (7.7)	89,697 (10.4)
Sex												
Male	35,314 (55.6)	34,911 (55.3)	35,838 (55.3)	36,615 (55.0)	37,716 (54.8)	38,953 (55.0)	40,120 (55.1)	41,722 (55.4)	68,914 (58.9)	61,426 (57.1)	51,942 (56.5)	483,471 (56.1)
Female	28,197 (44.4)	28,193 (44.7)	28,932 (44.7)	29,983 (45.0)	31,132 (45.2)	31,888 (45.0)	32,669 (44.9)	33,633 (44.6)	48,116 (41.1)	46,222 (42.9)	40,012 (43.5)	378,977 (43.9)
Age Group												
0–4 years	2679 (4.2)	2600 (4.1)	2669 (4.1)	2717 (4.1)	2683 (3.9)	2779 (3.9)	2818 (3.9)	2725 (3.6)	2098 (1.8)	2153 (2.0)	2249 (2.4)	28,170 (3.3)
5–9 years	1281 (2.0)	1186 (1.9)	1099 (1.7)	1154 (1.7)	1215 (1.8)	1151 (1.6)	1193 (1.6)	1126 (1.5)	954 (0.82)	1017 (0.9)	1111 (1.2)	12,487 (1.5)
10–14 years	854 (1.3)	857 (1.4)	809 (1.2)	838 (1.3)	837 (1.2)	808 (1.1)	818 (1.1)	827 (1.1)	735 (0.6)	849 (0.8)	852 (0.9)	9084 (1.1)
15–19 years	1444 (2.3)	1341 (2.1)	1237 (1.9)	1270 (1.9)	1275 (1.9)	1246 (1.8)	1288 (1.8)	1276 (1.7)	1157 (1.0)	1309 (1.2)	1531 (1.7)	14,374 (1.7)
20–24 years	1875 (3.0)	1759 (2.8)	1733 (2.7)	1750 (2.6)	1663 (2.4)	1737 (2.5)	1695 (2.3)	1833 (2.4)	1825 (1.6)	2223 (2.1)	2686 (2.9)	20,779 (2.4)
25–29 years	1924 (3.0)	1753 (2.8)	1776 (2.7)	1701 (2.6)	1657 (2.4)	1725 (2.4)	1756 (2.4)	1910 (2.5)	1983 (1.7)	2430 (2.3)	2998 (3.3)	21,613 (2.5)
30–34 years	1807 (2.8)	1737 (2.8)	1698 (2.6)	1616 (2.4)	1716 (2.5)	1629 (2.3)	1671 (2.3)	1826 (2.4)	2096 (1.8)	2514 (2.3)	2674 (2.9)	20,984 (2.4)
35–39 years	1636 (2.6)	1659 (2.6)	1703 (2.6)	1708 (2.6)	1794 (2.6)	1750 (2.5)	1871 (2.6)	1887 (2.5)	2445 (2.1)	2683 (2.5)	2584 (2.8)	21,740 (2.5)
40–44 years	1833 (2.9)	1743 (2.8)	1840 (2.8)	1805 (2.7)	1854 (2.7)	1812 (2.6)	1912 (2.6)	2065 (2.7)	3051 (2.6)	3150 (2.9)	2809 (3.1)	23,874 (2.8)
45–49 years	2167 (3.4)	2107 (3.3)	2193 (3.4)	2104 (3.2)	2215 (3.2)	2231 (3.2)	2325 (3.2)	2427 (3.2)	3795 (3.2)	3724 (3.5)	2917 (3.2)	29,205 (3.3)
50–54 years	2707 (4.3)	2600 (4.1)	2705 (4.2)	2707 (4.1)	2883 (4.2)	2836 (4.0)	2942 (4.0)	2922 (3.9)	5142 (4.4)	4878 (4.5)	3585 (3.9)	35,907 (4.2)
55–59 years	3237 (5.1)	3261 (5.2)	3306 (5.1)	3351 (5.0)	3579 (5.2)	3621 (5.1)	3804 (5.2)	3872 (5.1)	7418 (6.3)	6567 (6.1)	4670 (5.1)	46,686 (5.4)
60–64 years	3803 (6.0)	3776 (6.0)	4051 (6.3)	4180 (6.3)	4463 (6.5)	4415 (6.2)	4602 (6.3)	4713 (6.3)	9743 (8.3)	8396 (7.8)	5646 (6.1)	57,788 (6.7)
65–69 years	4292 (6.8)	4431 (7.0)	4538 (7.0)	4794 (7.2)	5031 (7.3)	5130 (7.2)	5293 (7.3)	5611 (7.5)	11,501 (9.8)	9773 (9.1)	6887 (7.5)	67,281 (7.8)
70–74 years	5121 (8.1)	5091 (8.1)	5251 (8.1)	5422 (8.1)	5651 (8.2)	5957 (8.4)	6067 (8.3)	6311 (8.4)	12,189 (10.4)	10,524 (9.8)	7719 (8.4)	75,303 (8.7)
75–79 years	6067 (9.6)	6238 (9.9)	6062 (9.4)	6537 (9.8)	6507 (9.5)	6820 (9.6)	6923 (9.5)	6953 (9.2)	12,599 (10.8)	10,850 (10.1)	8493 (9.2)	84,049 (9.8)
≥80 years	20,784 (32.7)	20,915 (33.2)	22,066 (34.1)	22,922 (34.4)	23,818 (34.6)	25,177 (35.5)	25,751 (35.4)	26,944 (35.8)	38,277 (32.7)	34,558 (32.1)	32,418 (35.3)	293,630 (34.1)

Missing information	–	50 (<0.1)	34 (<0.1)	22 (<0.1)	7 (<0.1)	17 (<0.1)	60 (<0.1)	127 (0.2)	22 (<0.1)	50 (<0.1)	125 (0.14)	514 (<0.1)
Province of Death												
Azuay	3527 (5.6)	3548 (5.6)	3836 (5.9)	3828 (5.7)	3809 (5.5)	3952 (5.6)	4119 (5.7)	4100 (5.4)	5235 (4.5)	5514 (5.1)	4760 (5.2)	46,228 (5.4)
Bolivar	831 (1.3)	802 (1.3)	834 (1.3)	827 (1.2)	803 (1.2)	807 (1.1)	861 (1.2)	853 (1.1)	1111 (0.9)	1173 (1.1)	984 (1.1)	9886 (1.2)
Canar	1030 (1.6)	926 (1.5)	1031 (1.6)	1070 (1.6)	1040 (1.5)	1084 (1.5)	1130 (1.6)	1169 (1.6)	1555 (1.3)	1510 (1.4)	1336 (1.5)	12,881 (1.5)
Carchi	588 (0.9)	603 (1.0)	666 (1.0)	661 (1.0)	645 (0.9)	651 (0.9)	711 (1.0)	760 (1.0)	986 (0.8)	1086 (1.0)	849 (0.9)	8206 (1.0)
Cotopaxi	1751 (2.8)	1723 (2.7)	1760 (2.7)	1668 (2.5)	1718 (2.5)	1464 (2.1)	1858 (2.6)	1934 (2.6)	2606 (2.2)	2721 (2.5)	2177 (2.4)	21,380 (2.5)
Chimborazo	2338 (3.7)	2287 (3.6)	2326 (3.6)	2323 (3.5)	2312 (3.4)	2512 (3.5)	2430 (3.3)	2451 (3.3)	3461 (3.0)	3339 (3.1)	2773 (3.0)	28,552 (3.3)
El Oro	2461 (3.9)	2446 (3.9)	2511 (3.9)	2713 (4.1)	2709 (3.9)	2919 (4.1)	2994 (4.1)	3131 (4.2)	5126 (4.4)	5113 (4.7)	4036 (4.4)	36,159 (4.2)
Esmeraldas	1882 (3.0)	1607 (2.5)	1523 (2.4)	1596 (2.4)	1472 (2.1)	1537 (2.2)	1619 (2.2)	1804 (2.4)	2466 (2.1)	2649 (2.5)	2458 (2.7)	20,613 (2.4)
Guayas	18,022 (28.4)	18,382 (29.1)	18,832 (29.1)	19,264 (28.9)	20,114 (29.2)	20,748 (29.3)	20,259 (27.8)	21,654 (28.7)	38,892 (33.2)	30,032 (27.9)	27,418 (29.8)	253,617 (29.4)
Imbabura	1824 (2.9)	1702 (2.7)	1832 (2.8)	1810 (2.7)	1860 (2.7)	2041 (2.9)	1975 (2.7)	2046 (2.7)	2722 (2.3)	2921 (2.7)	2527 (2.7)	23,260 (2.7)
Loja	2123 (3.3)	2067 (3.3)	2116 (3.3)	2215 (3.3)	2214 (3.2)	2344 (3.3)	2476 (3.4)	2481 (3.3)	2962 (2.5)	3580 (3.3)	2844 (3.1)	27,422 (3.2)
Los Rios	2963 (4.7)	3033 (4.8)	3165 (4.9)	3173 (4.8)	3423 (5.0)	3553 (5.0)	3696 (5.1)	3712 (4.9)	5356 (4.6)	5535 (5.1)	4872 (5.3)	42,481 (4.9)
Manabi	5573 (8.8)	5614 (8.9)	5599 (8.6)	6100 (9.2)	6683 (9.7)	6301 (8.9)	6483 (8.9)	6797 (9.0)	10,730 (9.2)	9620 (8.9)	8721 (9.5)	78,221 (9.1)
Morona Santiago	388 (0.6)	410 (0.6)	446 (0.7)	449 (0.7)	458 (0.7)	481 (0.7)	526 (0.7)	534 (0.7)	683 (0.6)	688 (0.6)	643 (0.7)	5706 (0.7)
Napo	402 (0.6)	338 (0.5)	345 (0.5)	353 (0.5)	330 (0.5)	361 (0.5)	374 (0.5)	413 (0.5)	566 (0.5)	595 (0.6)	487 (0.5)	4564 (0.5)
Pastaza	245 (0.4)	215 (0.3)	254 (0.4)	262 (0.4)	305 (0.4)	289 (0.4)	372 (0.5)	356 (0.5)	445 (0.4)	475 (0.4)	406 (0.4)	3624 (0.4)
Pichincha	11,330 (17.8)	11,179 (17.7)	11,197 (17.3)	11,412 (17.1)	11,951 (17.4)	12,674 (17.9)	13,233 (18.2)	13,355 (17.7)	19,727 (16.9)	19,621 (18.2)	15,077 (16.4)	150,756 (17.5)
Tungurahua	2628 (4.1)	2566 (4.1)	2599 (4.0)	2637 (4.0)	2698 (3.9)	2537 (3.6)	2828 (3.9)	2834 (3.8)	4177 (3.6)	4132 (3.8)	3166 (3.4)	32,802 (3.8)
Zamora Chinchipe	225 (0.4)	250 (0.4)	234 (0.4)	237 (0.4)	212 (0.3)	249 (0.4)	252 (0.3)	276 (0.4)	384 (0.3)	442 (0.4)	394 (0.4)	3155 (0.4)
Galapagos	25 (<0.1)	25 (<0.1)	37 (<0.1)	39 (<0.1)	53 (<0.1)	43 (<0.1)	53 (<0.1)	47 (<0.1)	53 (<1.0)	71 (<0.1)	64 (<0.1)	510 (0.1)
Sucumbios	505 (0.8)	537 (0.9)	580 (0.9)	542 (0.8)	567 (0.8)	612 (0.9)	636 (0.9)	673 (0.9)	962 (0.8)	1003 (0.9)	827 (0.9)	7444 (0.9)
Orellana	366 (0.6)	360 (0.6)	356 (0.5)	402 (0.6)	349 (0.5)	383 (0.5)	422 (0.6)	445 (0.6)	586 (0.5)	629 (0.6)	579 (0.6)	4877 (0.6)
Santo Domingo de los Tsachilas	1402 (2.2)	1423 (2.3)	1547 (2.4)	1769 (2.7)	1911 (2.8)	1981 (2.8)	2131 (2.9)	2196 (2.9)	3239 (2.8)	3274 (3.0)	28,48 (3.1)	23,721 (2.8)
Santa Elena	1060 (1.7)	1049 (1.7)	1114 (1.7)	1231 (1.8)	1209 (1.8)	1310 (1.8)	1349 (1.9)	1334 (1.8)	3000 (2.6)	1925 (1.8)	1708 (1.9)	16,289 (1.9)
Exterior	2 (<0.1)	3 (<0.1)	5 (<0.1)	3 (<0.1)	1 (<0.1)	7 (<0.1)	2 (<0.1)	–	–	–	–	23 (<0.1)

Undelimited Zones	20 (<0.1)	9 (<0.1)	25 (<0.1)	14 (<0.1)	2 (<0.1)	1 (<0.1)	–	–	–	–	–	71 (<0.1)
Area of Residence												
Urban	50,635 (79.7)	49,912 (79.1)	50,067 (77.3)	50,975 (76.5)	53,086 (77.1)	543,29 (76.7)	55,756 (76.6)	57,864 (76.8)	92,744 (79.2)	83,113 (77.2)	70,081 (76.2)	668,562 (77.5)
Rural	12,859 (20.2)	13,192 (20.9)	14,703 (22.7)	15,623 (23.5)	15,762 (22.9)	16,512 (23.3)	17,033 (23.4)	17,491 (23.2)	24,286 (20.8)	24,535 (22.8)	21,873 (23.8)	193,869 (22.5)
Missing information	17 (<0.1)	–	–	–	–	–	–	–	–	–	–	17 (<0.1)
Level of Education												
No formal education	14,933 (23.5)	14,908 (23.6)	2063 (3.2)	1930 (2.9)	15,201 (22.1)	37,291 (52.6)	13,173 (18.1)	32,151 (42.7)	18,972 (16.2)	18,044 (16.8)	15,648 (17.0)	184,314 (21.4)
Primary/Basic	26,420 (41.6)	26,575 (42.1)	28,181 (43.5)	28,858 (43.3)	30,253 (43.9)	15,178 (21.4)	33,473 (46.0)	21,321 (28.3)	55,107 (47.1)	46,174 (42.9)	42,579 (46.3)	354,119 (41.1)
Secondary and above	11,897 (18.7)	12,202 (19.3)	13,052 (20.2)	13,189 (19.8)	14,108 (20.5)	12,193 (17.2)	15,707 (21.6)	15,691 (20.8)	28,736 (24.6)	33,177 (30.8)	22,477 (24.4)	192,429 (22.3)
Child aged <20 years	6258 (9.9)	5984 (9.5)	5814 (9.0)	5979 (9.0)	6010 (8.7)	5984 (8.6)	6117 (8.4)	5954 (7.9)	4944 (4.2)	5328 (5.0)	5743 (6.3)	64,115 (7.4)
Missing information	4003 (6.3)	3435 (5.4)	15,660 (24.2)	16,642 (25.0)	3276 (4.8)	195 (0.3)	4319 (5.9)	238 (0.3)	9271 (7.9)	4925 (4.6)	5507 (6.0)	67,471 (7.8)
Ethnicity												
Indigenous	No data	3717 (5.9)	3822 (5.9)	3663 (5.5)	3674 (5.3)	3783 (5.3)	3715 (5.1)	3978 (5.3)	4815 (4.1)	4562 (4.2)	4300 (4.7)	40,029 (4.6)
Afro-Ecuadorian/Afro-Ecuadorian descent	No data	752 (1.2)	678 (1.0)	693 (1.0)	720 (1.0)	741 (1.0)	735 (1.0)	781 (1.0)	960 (0.8)	1046 (1.0)	1190 (1.3)	8296 (1.0)
Black	No data	590 (0.9)	465 (0.7)	574 (0.9)	558 (0.8)	488 (0.7)	546 (0.8)	569 (0.8)	647 (0.6)	632 (0.6)	529 (0.6)	5598 (0.7)
Mulato	No data	482 (0.8)	533 (0.8)	419 (0.6)	455 (0.7)	371 (0.5)	379 (0.5)	345 (0.5)	464 (0.4)	367 (0.3)	325 (0.4)	4140 (0.5)
Montubio	No data	1253 (2.0)	1357 (2.1)	1445 (2.2)	1670 (2.4)	1850 (2.6)	1994 (2.7)	1948 (2.6)	3091 (2.6)	2596 (2.4)	2511 (2.7)	19,715 (2.3)
Mestizo	No data	50,119 (79.4)	53,061 (81.9)	54,160 (81.3)	56,521 (82.1)	58,173 (82.1)	59,823 (82.2)	61,628 (81.8)	95,623 (81.7)	92,653 (86.1)	77,698 (84.5)	659,459 (76.5)
White	No data	1380 (2.2)	1547 (2.4)	1596 (2.4)	1578 (2.3)	1452 (2.0)	1234 (1.7)	1313 (1.7)	1539 (1.3)	1096 (1.0)	1120 (1.2)	13,855 (1.6)
Other	No data	210 (0.3)	121 (0.2)	79 (0.1)	80 (0.1)	109 (0.2)	93 (0.1)	124 (0.2)	120 (0.1)	148 (0.1)	144 (0.2)	1228 (0.1)
Missing information	63,511 (100.0)	4601 (7.3)	3186 (4.9)	3969 (6.0)	3592 (5.2)	3874 (5.5)	4270 (5.9)	4669 (6.2)	9771 (8.3)	4548 (4.2)	4137 (4.5)	110,128 (12.8)
Place of Death												
Establishments of the Ministry of Public Health	10,944 (17.2)	11,877 (18.8)	13,153 (20.3)	14,143 (21.2)	14,920 (21.7)	16,000 (22.6)	17,389 (23.9)	18,163 (24.1)	27,620 (23.6)	26,018 (24.2)	20,287 (22.1)	190,514 (22.9)
IESS establishments	4165 (6.6)	4947 (7.8)	5376 (8.3)	5885 (8.8)	6623 (9.6)	6929 (9.8)	8013 (11.0)	8337 (11.1)	13,923 (11.9)	14,165 (13.2)	9084 (9.9)	87,447 (10.1)
Non-Governmental Organization private hospital	1350 (2.1)	2183 (3.5)	2069 (3.2)	1934 (2.9)	1780 (2.6)	1798 (2.5)	1392 (1.9)	1428 (1.9)	1439 (1.2)	1468 (1.4)	1206 (1.3)	18,047 (2.1)
Other public establishment	–	–	1766 (2.7)	–	1825 (2.7)	520 (0.7)	650 (0.9)	658 (0.9)	1083 (0.9)	1174 (1.1)	640 (0.7)	8316 (1.0)

Private hospital	6804 (10.7)	5615 (8.9)	5274 (8.1)	5133 (7.7)	4629 (6.7)	6967 (9.8)	7194 (9.9)	7339 (9.7)	8862 (7.6)	10511 (9.8)	8498 (9.2)	76,826 (8.9)
Home	31,636 (49.8)	29,993 (47.5)	31,104 (48.0)	31,848 (47.8)	33,791 (49.1)	33,499 (47.3)	33,529 (46.1)	34,673 (46.0)	58,149 (49.7)	4,8120 (44.7)	44,160 (48.0)	410,502 (47.6)
Other	8612 (13.6)	8489 (13.5)	6028 (9.3)	7655 (11.5)	5280 (7.7)	5128 (7.2)	4622 (6.3)	4757 (6.3)	4288 (3.7)	5846 (5.4)	7839 (8.5)	68,544 (8.0)
Missing information	–	–	–	–	–	–	–	–	1666 (1.4)	346 (0.3)	240 (0.3)	2252 (0.3)
Climate Region of Death												
Coastal	31,961 (50.3)	32,131 (50.9)	32,744 (50.6)	34,077 (51.2)	35,610 (51.7)	36,368 (51.3)	36,400 (50.0)	38,432 (51.0)	65,570 (56.0)	54,874 (51.0)	49,213 (53.5)	447,380 (51.9)
Sierra	29,372 (46.2)	28,826 (45.7)	29,744 (45.9)	30,220 (45.4)	30,961 (45.0)	32,047 (45.2)	33,752 (46.4)	34,179 (45.4)	47,781 (40.8)	48,871 (45.4)	39,341 (42.8)	385,094 (44.7)
Amazon	2131 (3.4)	2110 (3.3)	2215 (3.4)	2245 (3.4)	2221 (3.2)	2375 (3.4)	2582 (3.5)	2697 (3.6)	3626 (3.1)	3832 (3.6)	3336 (3.6)	293,70 (3.4)
Insular/Galapagos	25 (<0.1)	25 (<0.1)	37 (<0.1)	39 (<0.1)	53 (0.1)	43 (<0.1)	53 (<0.1)	47 (<0.1)	53 (<0.1)	71 (<0.1)	64 (<0.1)	510 (<0.1)
Exterior/Undelimited zones/Other	22 (<0.1)	12 (<0.1)	30 (<0.1)	17 (<0.1)	3 (<0.1)	8 (<0.1)	2 (<0.1)	–	–	–	–	94 (<0.1)

Table S2: Number of lower respiratory infection (LRI) deaths in Ecuador by International Statistical Classification of Diseases and Related Health Problems, 10th Revision (ICD-10) code and year, 2012-2022. ICD-10 codes with no observations are not shown.

ICD-10 Code	Disease	2012	2013	2014	2015	2016	2017	2018	2019	2020	2021	2022	Total
	Total	3063	3252	3013	3026	3157	3481	3847	3858	30,568	25,311	7121	89,697
COVID-19	Coronavirus Disease	0	0	0	0	0	0	0	0	23,793	21,002	3279	48,074
J09.0	Influenza due to identified zoonotic or pandemic influenza virus	1	5	6	4	26	0	31	0	2	2	1	78
J10.0	Influenza due to identified seasonal influenza virus	2	23	3	1	8	2	5	5	0	10	1	60
J10.1	Influenza with other respiratory manifestations, seasonal influenza virus identified	0	1	1	0	2	0	0	0	0	0	0	4
J10.8	Influenza with other manifestations, seasonal influenza virus identified	1	2	0	1	0	0	0	0	0	0	0	4
J11.0	Influenza with pneumonia, virus not identified	6	9	15	5	21	20	40	7	17	59	6	205
J11.1	Influenza with other respiratory manifestations, virus not identified	11	20	26	23	16	8	7	3	13	6	9	142
J11.8	Influenza with other manifestations, virus not identified	2	3	0	0	0	0	0	1	3	0	1	10
J12.0	Adenoviral pneumonia	0	0	0	0	0	0	0	0	0	0	1	1
J12.1	Respiratory syncytial virus pneumonia	0	3	1	0	0	5	3	1	3	5	5	26
J12.2	Parainfluenza virus pneumonia	0	0	0	1	0	0	1	0	0	0	1	3
J12.3	Human metapneumovirus pneumonia	0	0	0	1	0	0	1	0	0	0	1	3
J12.8	Other viral pneumonia	0	0	0	0	0	0	0	1	47	14	2	64
J12.9	Viral pneumonia, unspecified	7	10	3	3	8	4	12	9	2406	719	144	3325
J13.0	Pneumonia due to Streptococcus pneumoniae	2	3	5	6	4	4	4	3	5	4	2	42
J14.0	Pneumonia due to Haemophilus influenzae	0	1	0	0	1	0	0	1	0	0	0	3
J15.0	Pneumonia due to Klebsiella pneumoniae	4	3	14	15	10	8	10	15	15	30	26	150
J15.1	Pneumonia due to Pseudomonas	2	5	5	5	2	4	4	8	6	5	6	52
J15.2	Pneumonia due to staphylococcus	1	7	7	3	3	7	5	8	0	3	6	50
J15.4	Pneumonia due to other streptococci	0	0	3	1	2	1	2	1	0	0	2	12
J15.5	Pneumonia due to Escherichia coli	0	1	2	1	2	0	1	0	0	2	1	10
J15.6	Pneumonia due to other Gram-negative bacteria	0	2	1	3	1	0	3	5	2	1	3	21
J15.7	Pneumonia due to Mycoplasma pneumoniae	2	0	0	0	1	0	0	0	0	0	0	3
J15.8	Other bacterial pneumonia	11	9	9	5	6	1	4	7	7	18	13	90
J15.9	Bacterial pneumonia, unspecified	178	308	443	668	689	674	746	761	646	856	798	6767
J16.8	Pneumonia due to other specified infectious organisms	3	0	1	1	1	0	1	2	10	8	4	31
J18.1	Lobar pneumonia, unspecified	40	36	22	29	18	10	18	18	13	29	27	260
J18.2	Hypostatic pneumonia, unspecified	6	2	7	13	11	9	32	27	26	36	27	196
J18.8	Other pneumonia, organism unspecified	297	1	3	1	1	1	2	6	3	2	7	324

J18.9	Pneumonia, unspecified	2215	2574	2285	2034	2115	2553	2739	2784	3292	2380	2618	27,589
J20.5	Acute bronchitis due to respiratory syncytial virus	0	0	0	1	0	0	0	0	0	0	0	1
J20.8	Acute bronchitis due to other specified organisms	0	0	0	0	1	0	0	0	0	0	0	1
J20.9	Acute bronchitis, unspecified	60	51	53	57	63	66	37	46	59	20	26	538
J21.8	Acute bronchiolitis due to other specified organisms	0	0	0	0	0	0	0	0	0	0	1	1
J21.9	Acute bronchiolitis, unspecified	22	7	8	7	9	12	7	4	8	3	2	89
J22.0	Unspecified acute lower respiratory infection	33	15	15	15	12	15	20	20	122	40	30	337
J85.1	Abscess of lung with pneumonia	0	5	2	8	10	4	4	7	3	6	6	55
P23.0	Congenital pneumonia due to viral agent	0	0	0	1	0	11	14	8	1	0	2	37
P23.1	Congenital pneumonia due to Chlamydia	2	0	0	1	0	0	0	1	0	0	0	4
P23.2	Congenital pneumonia due to staphylococcus	0	0	0	3	1	0	0	1	1	0	1	7
P23.3	Congenital pneumonia due to streptococcus, group B	0	0	0	0	1	0	0	0	0	0	0	1
P23.4	Congenital pneumonia due to Escherichia coli	0	1	0	1	0	0	0	0	0	0	0	2
P23.5	Congenital pneumonia due to Pseudomonas	0	2	0	0	0	0	0	0	0	0	0	2
P23.6	Congenital pneumonia due to other bacterial agents	1	6	3	9	3	3	3	5	1	0	1	35
P23.8	Congenital pneumonia due to other organisms	0	0	0	1	0	0	0	1	2	0	0	4
P23.9	Congenital pneumonia, unspecified	154	137	70	98	109	59	91	92	62	51	61	984

Table S3: Frequency of registered deaths in Ecuador, 2012–2019, by cause of death and socio-demographic data. Number (*N*) and column percents (%) are shown by lower respiratory infection (LRI) attributed death, LRI aetiology, and deaths from other causes.

Variable	Deaths from other causes *N* (%)	LRI (Total) *N* (%)	Influenza *N* (%)	Pneumonia, Viral *N* (%)	Pneumonia, bacterial *N* (%)	Pneumonia, unspecified *N* (%)	Bronchiolitis/ Other *N* (%)	Total deaths N (%)
Total Deaths	519, 119 (95.11)	26,697 (4.89)	373 (0.07)	108 (0.02)	4790 (0.88)	20,770 (3.81)	656 (0.12)	545,816 (100)
Sex								
Male	287,458 (55.37)	13,731 (51.43)	192 (51.47)	62 (57.41)	2468 (51.52)	10,678 (51.41)	331 (50.46)	301,189 (55.18)
Female	231,661 (44.63)	12,966 (48.57)	181 (48.53)	46 (42.59)	2322 (48.48)	10,092 (48.59)	325 (49.54)	244,627 (44.82)
Age Group								
0–4 years	19,628 (3.78)	2042 (7.65)	30 (8.04)	33 (30.56)	186 (3.88)	1618 (7.79)	175 (26.68)	21,670 (3.97)
5–14 years	14,861 (2.86)	1192 (4.46)	16 (4.29)	17 (15.74)	125 (2.61)	958 (4.61)	76 (11.59)	16,053 (2.94)
15–49 years	96,696 (18.63)	2269 (8.50)	70 (18.77)	14 (12.96)	367 (7.66)	1779 (8.57)	39 (5.95)	98,965 (18.13)
50–69 years	119,891 (23.10)	3565 (13.35)	98 (26.27)	15 (13.89)	780 (16.28)	2623 (12.63)	49 (7.47)	123,456 (22.62)
≥70 years	267,745 (51.58)	17,610 (65.96)	159 (42.62)	29 (26.85)	3,331 (69.54)	13,774 (66.32)	317 (48.32)	285,355 (52.28)
Missing information	298 (0.06)	19 (0.07)	–	–	1 (0.02)	18 (0.09)	–	317 (0.06)
Area of Residence								
Urban	401,574 (77.36)	21,050 (78.85)	254 (68.10)	82 (75.93)	3939 (82.23)	16324 (78.59)	451 (68.75)	42,262 (77.43)
Rural	117,528 (22.64)	5647 (21.15)	119 (31.90)	26 (24.07)	851 (17.77)	4446 (21.41)	205 (31.25)	123,175 (22.57)
Missing information	17 (<0.01)	–	–	–	–	–	–	17 (<0.01)
Level of Education								
No formal education	124,827 (24.05)	6823 (25.56)	72 (19.30)	18 (16.67)	1213 (25.32)	5358 (25.80)	162 (24.7)	131,650 (24.12)
Primary/Basic	200,742 (38.67)	9517 (35.65)	117 (31.37)	20 (18.52)	1792 (37.41)	7456 (35.90)	132 (20.12)	210,259 (38.52)
Secondary and above	103,475 (19.93)	4564 (17.10)	92 (24.66%)	13 (12.04)	1004 (20.96)	3412 (16.43)	43 (6.55)	108,039 (19.79)
Child aged <20 years	44,601 (8.59)	3499 (13.11)	50 (13.40)	52 (48.15)	348 (7.27)	2792 (13.44)	257 (39.18)	48,100 (8.81)
Missing information	45,474 (8.76)	2294 (8.59)	42 (11.26)	5 (4.63)	433 (9.04)	1752 (8.44)	62 (9.45)	47,768 (8.75)
Ethnicity								
Indigenous	24,934 (4.80)	1418 (5.31)	48 (12.87)	3 (2.78)	167 (3.49)	1128 (5.43)	72 (10.98)	26,352 (4.83)
Ecuadorian	374,191 (72.08)	19,294 (72.27)	262 (70.24)	81 (75.00)	3769 (78.68)	14,811 (71.31)	371 (56.55)	393,485 (72.09)
Other	32,936 (6.34)	1,371 (5.14)	18 (4.83)	3 (2.78)	302 (6.30)	984 (4.74)	64 (9.76)	34,307 (6.29)
Missing information	87,058 (16.77)	4614 (17.28)	45 (12.06)	21 (19.44)	552 (11.52)	3847 (18.52)	149 (22.71)	91,672 (16.80)
Place of Death								
Public hospital	159,866 (30.80)	12,417 (46.51)	152 (40.75)	46 (42.59)	2117 (44.20)	9982 (48.06)	120 (18.29)	172,283 (31.56)
Private hospital	57,938 (11.16)	4951 (18.55)	46 (12.33)	33 (30.56)	1233 (25.74)	3596 (17.31)	43 (6.55)	62,889 (11.52)
Home	251,543 (48.46)	8530 (31.95)	162 (43.43)	27 (25.00)	1345 (28.08)	6531 (31.44)	465 (70.88)	260,073 (47.65)
Other	49,772 (9.59)	799 (2.99)	13 (3.49)	2 (1.85)	95 (1.98)	661 (3.18)	28 (4.27)	50,571 (9.27)
Missing information	–	–	–	–	–	–	–	–
Climate Region of Death								
Coastal	264,713 (50.99)	13,010 (48.73)	61 (16.35)	50 (46.30)	3089 (64.49)	9527 (45.87)	283 (43.14)	277,723 (50.88)
Sierra	236,004 (45.46)	13,097 (49.06)	304 (81.50)	55 (50.93)	1634 (34.11)	10,748 (51.75)	356 (54.27)	249,101 (45.64)
Amazon	17,993 (3.47)	583 (2.18)	8 (2.14)	3 (2.78)	64 (1.34)	491 (2.36)	17 (2.59)	18,576 (3.40)

Insular/Galapagos	316 (0.06)	6 (0.02)	–	–	3 (0.06)	3 (0.01)	–	322 (0.06)
Exterior/Undelimited zones	93 (0.02)	1 (<0.01)	–	–	–	1 (<0.01)	–	94 (0.02)

Table S4: Frequency of registered deaths in Ecuador, 2020–2022, by cause of death and socio-demographic data. Number (*N*) and column percents (%) are shown by lower respiratory infection (LRI) attributed death, LRI aetiology, and deaths from other causes.

Variable	Deaths from other causes *N* (%)	LRI (Total) *N* (%)	COVID-19 *N* (%)	Influenza *N* (%)	Pneumonia, Viral *N* (%)	Pneumonia, bacterial *N* (%)	Pneumonia, unspecified *N* (%)	Bronchiolitis/ Other *N* (%)	Total deaths *N* (%)
Total Deaths	253,632 (80.10)	63,000 (19.90)	48,074 (15.18)	130 (0.04)	3351 (1.06)	2461 (0.78)	8673 (2.74)	311 (0.10)	316,632 (100)
Sex									
Male	143,127 (56.43)	39,155 (62.15)	30,393 (63.22)	63 (48.46)	2191 (65.38)	1393 (56.60)	4941 (56.97)	174 (55.95)	182,282 (57.57)
Female	110,505 (43.57)	23,845 (37.85)	17,681 (36.78)	67 (51.54)	1160 (34.62)	1068 (43.40)	3732 (43.03)	137 (44.05)	134,350 (42.43)
Age Group									
0–4 years	5925 (2.34)	575 (0.91)	100 (0.21)	4 (3.08)	18 (0.54)	43 (1.75)	381 (4.39)	29 (9.32)	6500 (2.05)
5–14 years	5069 (2.00)	449 (0.71)	117 (0.24)	3 (2.31)	12 (0.36)	37 (1.50)	270 (3.11)	10 (3.222)	5518 (1.74)
15–49 years	46,393 (18.30)	6191 (9.84)	4737 (9.85)	18 (13.85)	289 (8.62)	222 (9.02)	907 (10.46)	18 (5.79)	52,584 (16.61)
50–69 years	60,720 (23.95)	23,486 (37.20)	19,535 (40.64)	45 (34.62)	1342 (40.05)	518 (21.05)	1962 (22.62)	84 (27.01)	84,206 (26.59)
≥70 years	135,395 (53.41)	32,232 (51.22)	23,522 (48.93)	60 (46.15)	1690 (50.43)	1641 (66.68)	5149 (59.37)	170 (54.66)	167,627 (52.94)
Missing information	130 (0.05)	67 (0.11)	63 (0.13)	–	–	–	4 (0.05)	–	197 (0.06)
Area of Residence									
Urban	193,889 (76.45)	52,049 (82.62)	39,685 (82.55)	104 (80.00)	3169 (94.57)	1933 (78.55)	6930 (79.90)	228 (73.31)	245,938 (77.67)
Rural	59,743 (23.55)	10,951 (17.38)	8389 (17.45)	26 (20.00)	182 (5.43)	528 (21.45)	1743 (20.10)	83 (26.69)	70,694 (22.33)
Missing information	–	–	–	–	–	–	–	–	–
Level of Education									
No formal education	45,906 (18.10)	6758 (10.73)	4506 (9.37)	30 (23.08)	272 (8.12)	410 (16.66)	1473 (17.98)	67 (21.54)	52,664 (16.63)
Primary/Basic	111,232 (43.86)	32,628 (51.79)	25,969 (54.02)	67 (51.54)	1600 (47.75)	1122 (45.59)	3746 (43.19)	124 (39.87)	143,860 (45.43)
Secondary and above	67,700 (26.69)	16,690 (26.49)	12,903 (26.84)	21 (16.15)	985 (29.39)	692 (28.12)	2025 (23.35)	64 (20.58)	84,390 (26.65)
Child aged <20 years	14,805 (5.84)	1210 (1.92)	286 (0.59)	7 (5.38)	38 (1.13)	105 (4.27)	734 (8.46)	40 (12.86)	16,015 (5.06)
Missing information	13,989 (5.52)	5714 (9.07)	4410 (9.17)	5 (3.85)	456 (13.61)	132 (5.36)	695 (8.01)	16 (5.14)	19,703 (6.22)
Ethnicity									
Indigenous	12,140 (4.79)	1537 (2.44)	943 (1.96)	8 (6.15)	38 (1.13)	89 (3.62)	433 (4.99)	26 (8.36)	13,677 (4.32)
Ecuadorian	211,584 (83.42)	54,390 (86.33)	41,944 (87.2%)	118 (90.77)	2768 (82.60)	2150 (87.36)	7174 (82.72)	236 (75.88)	265,974 (84.00)
Other	16,492 (6.50)	2033 (3.23)	1398 (2.91)	2 (1.54)	114 (3.40)	95 (3.86)	401 (4.62)	23 (7.40)	18,525 (5.85)
Missing information	13,416 (5.29)	5040 (8.00)	3789 (7.88)	2 (1.54)	431 (12.86)	127 (5.16)	665 (7.67)	26 (8.36)	18,456 (5.83)
Place of Death									
Public hospital	68,851 (27.15)	45,143 (71.66)	38,101 (79.25)	96 (73.85)	1423 (42.46)	1107 (44.98)	4314 (49.74)	102 (32.80)	113,994 (36.00)
Private hospital	25,081 (9.89)	6903 (10.96)	4443 (9.24)	7 (5.38)	484 (14.44)	603 (24.50)	1341 (15.46)	25 (8.04)	31,984 (10.10)
Home	140,396 (55.35)	10,033 (15.9%)	4,882 (10.16)	24 (18.46)	1417 (42.29)	715 (29.05)	2818 (32.49)	177 (56.91)	150,429 (47.51)
Other	17,572 (6.93)	401 (0.64)	245 (0.51)	3 (2.31)	6 (0.18)	13 (0.53)	133 (1.53)	1 (0.32)	17,973 (5.68)
Missing information	1732 (0.68)	520 (0.83)	403 (0.84)	–	21 (0.63)	23 (0.93)	67 (0.77)	6 (1.93)	2252 (0.71)
Climate Region of Death									
Coastal	137,253 (54.12)	32,404 (51.43)	22,758 (47.34)	25 (19.23)	3029 (90.4%)	1412 (57.38)	4963 (57.22)	217 (69.77)	169,657 (53.58)
Sierra	107,161 (42.25)	28,832 (45.77)	23,883 (49.68)	100 (76.92)	308 (9.19)	1009 (41.00)	3443 (39.70)	89 (28.62)	135,993 (42.95)
Amazon	9066 (3.57)	1728 (2.74)	1403 (2.92)	5 (3.85)	13 (0.39)	37 (1.50)	265 (3.06)	5 (1.61)	10,794 (3.41)

Insular/Galapagos	152 (0.06)	36 (0.06)	30 (0.06)	–	1 (<0.01)	3 (0.03)	2 (0.12)	–	188 (0.06)
Exterior/Undelimited zones	–	–	–	–	–	–	–	–	–

Table S5: Age-specific lower respiratory infection (LRI) mortality trend analysis, 2012–2022**.** Average annual percentage change (AAPC) is calculated for 2012–2022, and annual percentage change (APC) is calculated for each trend segment. 95% Confidence intervals (CI) are shown. *N*=514 are missing from age-specific analysis.

	2012–2022								
	**AAPC (95% CI)**[1]	**Trend 1**[1]		**Trend 2**[1]		**Trend 3**[1]		**Trend 4**[1]	
**Age Group**	**2012–2022**	**Years**	**APC (95% CI)**	**Years**	**APC (95% CI)**	**Years**	**APC (95% CI)**	**Years**	**APC (95% CI)**
Male									
All ages	12.34 (−0.33, 29.12)	2012–2017	−10.50 (−71.47, 19.99)	2017–2020	156.90 (77.36, 295.51)	2020–2022	−42.66 (−67.75, −14.16)	–	–
0–4 years	−3.28 (−5.89, −0.77)	–	–	–	–	–	–	–	–
5–14 years	−1.26 (−3.79, 1.96)	2012–2014	−12.25 (−34.57, 2.22)	2014–2022	1.69 (−20.22, 25.94)	–	–	–	–
15–49 years	14.54 (ND, ND)	2012–2021	43.35 (−27.72, 17683.80)	2021–2022	−83.80 (−99.53, 107.47)	–	–	–	–
50–69 years	19.92 (1.65, 51.88)	2012–2017	−17.05 (−84.77, 34.74)	2017–2020	293.04 (152.84, 696.75)	2020–2022	−49.21 (−71.30, −21.64)	–	–
≥70 years	8.77 (−2.44, 22.73)	2012–2017	−10.30 (−72.53, 16.37)	2017–2020	119.77 (56.10, 218.64)	2020–2022	−38.69 (−66.93, −10.97)		
Female									
All ages	5.76 (ND, ND)	2012–2017	−2.13 (−63.15, 19.26)	2017–2021	67.31 (39.67, 135.06)	2021–2022	−75.13 (−89.66, −34.74)	–	–
0–4 years	−2.50 (ND, ND)	2012–2015	−11.14 (−15.58, −8.78)	2015–2018	6.43 (3.04, 9.32)	2018–2021	−14.74 (−17.51, −12.10)	2021–2022	48.07 (34.12, 63.20)
5–14 years	−4.11 (−7.31, −0.05)	2012–2014	−23.61 (−48.16, −5.31)	2014–2022	1.49 (−2.28, 19.48)				
15–49 years	11.89 (ND, ND)	2012–2021	33.36 (−40.99, 6363.12)	2021–2022	−76.90 (−98.78, 93.29)	–	–	–	–
50–69 years	23.03 (ND, ND)	2012–2021	57.02 (43.23, 106,596.88)	2021–2022	−86.31 (−99.67, 64.09)	–	–	–	–
≥70 years	4.40 (ND, ND)	2012–2018	2.39 (−43.99, 14.34)	2021–2022	−69.04 (−83.47, −31.21)	–	–	–	–

[1] estimated by Joinpoint Regression model.

We were unable to accurately model trends between 2012–2022 using Joinpoint Regression Analysis software v5.2 due to the large increase in cases in 2020. This is reflected by high uncertainty in the estimates indicated by wide 95% CI. As the AAPC is calculated from the weighted APC of each trend, the AAPC does not accurately reflect the average percent change per year for these age groups. ND= not determined.

Table S6: Lower respiratory infection (LRI) age-standardized mortality rates (ASMR) per 100,000 population per province, 2012–2020. Percent change (%) was calculated 2012–2019 and 2019–2020. Average LRI ASMR was calculated from 2012–2019.

**Province**	**Year**									**Percent change (%)**		**Average ASMR**
	**2012**	**2013**	**2014**	**2015**	**2016**	**2017**	**2018**	**2019**	**2020**	**2012–2019**	**2019–2020**	**2012–2019**
National	22.22	22.96	20.76	20.23	20.45	21.87	23.29	22.45	173.71	1.04	673.76	21.78
Azuay	24.14	23.58	19.44	19.91	15.10	27.66	27.69	24.64	131.95	2.04	435.59	22.77
Bolivar	19.37	15.48	12.07	12.25	13.98	10.64	14.69	14.83	64.90	−23.45	337.58	14.16
Canar	12.74	13.79	9.91	6.31	4.87	12.68	15.35	15.72	119.80	23.40	662.12	11.42
Carchi	10.16	10.89	12.32	14.28	8.21	9.76	16.28	13.52	87.37	33.08	546.30	11.93
Cotopaxi	21.48	17.26	17.38	22.55	22.88	20.85	26.28	26.05	113.12	21.30	334.23	21.84
Chimborazo	28.97	33.63	35.03	24.37	29.11	25.24	26.32	28.29	138.14	−2.35	388.39	28.87
El Oro	18.28	17.28	16.07	22.10	19.66	40.63	39.82	39.57	256.62	116.42	548.61	26.68
Esmeraldas	8.51	10.33	9.41	10.87	7.73	8.93	13.86	14.84	124.02	74.40	735.84	10.56
Guayas	29.82	31.76	31.37	30.82	32.39	30.94	28.64	31.49	220.99	5.60	601.80	30.90
Imbabura	16.03	10.89	10.75	10.92	10.77	14.72	20.00	18.97	110.66	18.35	483.39	14.13
Loja	20.92	16.17	16.60	11.67	7.76	17.20	30.12	19.03	89.62	−9.03	370.91	17.44
Los Rios	7.23	7.46	7.04	8.63	7.86	11.43	17.13	16.18	151.26	123.73	834.83	10.37
Manabi	6.48	10.45	6.98	9.64	14.92	12.85	12.58	16.08	176.53	148.23	998.14	11.25
Morona Santiago	20.89	15.64	9.86	8.35	12.09	6.84	17.88	8.92	93.81	−57.32	952.05	12.56
Napo	19.29	6.30	17.23	13.67	22.95	7.30	14.54	27.27	135.26	41.38	396.06	16.07
Pastaza	14.89	22.81	10.63	11.95	21.46	13.21	23.25	23.80	178.72	59.81	650.85	17.75
Pichincha	21.77	25.17	21.52	18.43	19.82	19.00	24.49	22.23	185.37	2.12	733.79	21.56
Tungurahua	24.73	23.34	20.80	25.36	24.04	26.46	26.51	22.01	181.55	−11.02	724.95	24.16
Zamora Chinchipe	13.38	7.75	9.03	9.43	8.58	17.19	8.67	10.31	89.03	−22.91	763.21	10.54

Galapagos	6.49	0.00	4.88	6.03	2.74	11.15	0.00	0.00	68.64	−100	100	3.91
Sucumbios	7.43	12.20	14.20	11.00	6.39	4.83	11.61	9.56	163.81	28.69	1613.43	9.65
Orellana	6.59	15.37	7.65	6.48	3.18	8.81	5.50	34.26	105.58	419.54	208.19	10.98
Santo Domingo de los Tsachilas	12.42	11.54	10.61	19.51	19.67	24.35	29.83	38.30	247.26	208.27	545.56	20.78
Santa Elena	10.20	22.10	13.99	8.38	8.79	9.79	7.63	8.20	238.58	−19.60	2810.19	11.13
Undelimited Zones	–	–	–	3.77	–	–	–	–	–	–	–	0.47

Table S7: Association of sociodemographic risk factors and lower respiratory infection (LRI) mortality in Ecuador, 2012. Unadjusted odds ratio (OR) and adjusted odds ratio (aOR), 95% confidence intervals (CI) and *p* values are shown. Multivariable model includes sex, age group, area of residence, level of education, place of death and climate region of death. Ethnicity was excluded from the model for 2012, due to no data. Associations with LRI mortality are compared with those for deaths from other causes.

		**Unadjusted 2012**		**Adjusted 2012**	
	***N* (%)**	**OR (95% CI)**	***p* value**	**OR (95% CI)**	***p* value**
Sex					
Male	35,314 (55.60)	Reference			
Female	28,197 (44.40)	1.22 (1.14, 1.31)	<0.001	1.09 (1.01, 1.18)	0.035
Age Group (years)					
0–4	2679 (4.22)	5.14 (4.48, 5.90)	<0.001	1.87 (1.56, 2.24)	<0.001
5–69	28,860 (45.44)	Reference			
≥70	31,972 (50.34)	2.46 (2.26, 2.68)	<0.001	3.11 (2.80, 3.47)	<0.001
Area of Residence					
Urban	50,635 (79.73)	Reference			
Rural	12,859 (20.25)	0.91 (0.833, 1.00)	<0.001	0.99 (0.89, 1.09)	0.843
Ethnicity					
Indigenous	0	No data		No data	
Ecuadorian	0	Reference			
Other	0	No data		No data	
Level of Education					
No formal education	14,933 (25.09)	Reference			
Primary/Basic	26,420 (44.40)	0.90 (0.82, 0.99)	0.034	0.97 (0.88, 1.07)	0.576
Secondary and above	11,897 (19.99)	0.76 (0.67, 0.86)	<0.001	0.98 (0.86, 1.12)	0.747
Child aged <20 years	6258 (10.52)	1.75 (1.56, 1.96)	0.000	2.83 (2.35, 3.40)	0.000
Place of Death					
Public hospital	15,109 (23.79)	Reference			
Private hospital	8154 (12.84)	1.01 (1.92, 1.12)	0.784	0.97 (0.87, 1.09)	0.637
Home	31,636 (49.81)	0.44 (0.40, 0.48)	<0.001	0.40 (0.37, 0.44)	<0.001
Other/No information	8612 (13.56)	0.29 (0.25, 0.34)	<0.001	0.38 (0.32, 0.44)	<0.001
Climate Region of Death					
Coastal and Insular	32,008 (50.40)	Reference			
Sierra	29,372 (46.25)	1.29 (1.19, 1.38)	<0.001	1.19 (1.10, 1.29)	<0.001
Amazon	2131 (3.36)	0.82 (0.65, 1.04)	0.101	0.93 (0.73, 1.18)	0.530

Data are missing *N* (%) for Area of Residence 17 (0.03), Ethnicity 63,511 (100), Level of Education 4003 (6.30).

Table S8: Association of sociodemographic risk factors and lower respiratory infection (LRI) mortality in Ecuador, 2013. Unadjusted odds ratio (OR) and adjusted odds ratio (aOR), 95% confidence intervals (CI) and *p* values are shown. Multivariable model includes sex, age group, area of residence, ethnicity, level of education, place of death and climate region of death. Associations with LRI mortality are compared with those for deaths from other causes.

		Unadjusted 2013		Adjusted 2013	
	***N* (%)**	**OR (95% CI)**	***p* value**	**OR (95% CI)**	***p* value**
Sex					
Male	34,911 (55.32)	Reference			
Female	28,193 (44.68)	1.15 (1.07, 1.24)	<0.001	1.05 (0.97, 1.13)	0.249
Age Group (years)					
0–4	2600 (4.12)	3.43 (2.98, 3.95)	<0.001	1.60 (1.30, 1.96)	<0.001
5–69	28,210 (44.70)	Reference			
≥70	32,244 (51.10)	1.90 (1.76, 2.06)	<0.001	2.41 (2.19, 2.65)	<0.001
Area of Residence					
Urban	49,912 (79.09)	Reference			
Rural	13,192 (20.91)	0.88 (0.80, 0.96)	0.004	0.96 (0.87, 1.06)	0.439
Ethnicity					
Indigenous	3717 (5.89)	1.09 (0.94, 1.26)	0.261	1.39 (1.18, 1.64)	<0.001
Ecuadorian	50,119 (79.42)	Reference			
Other	4667 (7.40)	0.78 (0.67, 0.90)	0.001	0.84 (0.72, 0.98)	0.027
Level of Education					
No formal education	114,881 (23.58)	Reference			
Primary/Basic	26,457 (41.93)	0.95 (0.86, 1.04)	0.242	0.97 (0.88, 1.08)	0.605
Secondary and above	12,068 (19.12)	1.00 (0.90, 1.12)	0.983	1.16 (1.02, 1.31)	0.025
Child aged <20 years	6312 (10.0)	1.65 (1.47, 1.86)	<0.001	2.29 (1.89, 2.76)	<0.001
Place of Death					
Public hospital	16,824 (26.66)	Reference			
Private hospital	7,798 (12.36)	1.16 (1.06, 1.28)	0.002	1.09 (0.99, 1.21)	0.092
Home	29,993 (47.53)	0.37 (0.34, 0.40)	<0.001	0.32 (0.29, 0.35)	<0.001
Other/No information	8489 (13.45)	0.26 (0.23, 0.31)	<0.001	0.28 (0.24, 0.33)	<0.001
Climate Region of Death					
Coastal and Insular	32,168 (50.98)	Reference			
Sierra	28,826 (45.68)	1.18 (1.10, 1.27)	<0.001	1.13 (1.05, 1.23)	0.002
Amazon	2110 (3.34)	0.69 (0.54, 0.87)	0.002	0.74 (0.57, 0.97)	0.026

Data are missing *N* (%) Age Group 50 (0.08), Ethnicity 4601 (7.29), Level of Education 34356 (5.44).

Table S9: Association of sociodemographic risk factors and lower respiratory infection (LRI) mortality in Ecuador, 2014. Unadjusted odds ratio (OR) and adjusted odds ratio (aOR), 95% confidence intervals (CI) and *p* values are shown. Multivariable model includes sex, age group, area of residence, ethnicity, level of education, place of death and climate region of death. Associations with LRI mortality are compared with those for deaths from other causes.

		**Unadjusted 2014**		**Adjusted 2014**	
	N **(%)**	**OR (95% CI)**	***p*** **value**	**OR (95% CI)**	***p*** **value**
Sex					
Male	35,838 (55.33)	Reference			
Female	28,932 (44.67)	1.23 (1.14, 1.32)	<0.001	1.05 (0.96, 1.15)	0.279
Age Group (years)					
0–4	2669 (4.12)	3.46 (2.99, 4.02)	<0.001	1.81 (1.44, 2.27)	<0.001
5–69	28,688 (44.29)	Reference			
≥70	33,379 (51.53)	2.15 (1.97, 2.33)	<0.001	2.61 (2.34, 2.91)	<0.001
Area of Residence					
Urban	50,067 (77.30)	Reference			
Rural	14,703 (22.70)	0.94 (0.86, 1.02)	0.142	1.07 (0.95, 1.20)	0.290
Ethnicity					
Indigenous	3822 (5.90)	1.24 (1.07, 1.43)	0.004	1.84 (1.45, 2.33)	<0.001
Ecuadorian	53,061 (81.92)	Reference			
Other	4701 (7.26)	0.81 (0.69, 0.94)	0.007	0.98 (0.82, 1.16)	0.802
Level of Education					
No formal education	2063 (3.19)	Reference			
Primary/Basic	28,062 (43.33)	1.14 (0.91, 1.44)	0.261	1.16 (0.92, 1.48)	0.205
Secondary and above	12,863 (19.86)	1.11(0.87, 1.40)	0.408	1.25 (0.98, 1.61)	0.069
Child aged <20 years	6177 (9.54)	1.71 (1.33, 2.18)	<0.001	2.29 (1.69, 3.10)	<0.001
Place of Death					
Public hospital	20,295 (31.33)	Reference			
Private hospital	7343 (11.34)	1.34 (1.22, 1.48)	<0.001	1.32 (1.18, 1.48)	<0.001
Home	31,104 (48.02)	0.44 (0.40, 0.48)	<0.001	0.41 (0.37, 0.46)	<0.001
Other/No information	6028 (9.31)	0.13 (0.10, 0.17)	<0.001	0.15 (0.10, 0.20)	<0.001
Climate Region of Death					
Coastal and Insular	32,811 (50.66)	Reference			
Sierra	29,744 (45.92)	1.14 (1.06, 1.23)	0.001	0.99 (0.90, 1.08)	0.790
Amazon	2215 (3.42)	0.62 (0.48, 0.80)	<0.001	0.69 (0.50, 0.95)	0.022

Data are missing *N* (%) Age Group 34 (0.05), Ethnicity 3186 (4.92), Level of Education 15,660 (24.17).

Table S10: Association of sociodemographic risk factors and lower respiratory infection (LRI) mortality in Ecuador, 2015. Unadjusted odds ratio (OR) and adjusted odds ratio (aOR), 95% confidence intervals (CI) and *p* values are shown. Multivariable model includes sex, age group, area of residence, ethnicity, level of education, place of death and climate region of death. Associations with LRI mortality are compared with those for deaths from other causes.

		**Unadjusted 2015**		**Adjusted 2015**	
	***N* (%)**	**OR (95% CI)**	***p* value**	**OR (95% CI)**	***p* value**
Sex					
Male	36,615 (54.98)	Reference			
Female	29,983 (45.02)	1.20 (1.11, 1.29)	<0.001	1.05 (0.96, 1.15)	0.298
Age Group (years)					
0–4	2717 (4.08)	3.53 (3.03, 4.12)	<0.001	1.71 (1.37, 2.14)	<0.001
5–69	28,978 (43.51)	Reference			
≥70	34,881 (52.38)	2.39 (2.20, 2.61)	<0.001	3.18 (2.83, 3.57)	<0.001
Area of Residence					
Urban	50,975 (76.54)	Reference			
Rural	15,623 (23.46)	0.82 (0.75, 0.90)	<0.001	0.88 (0.78, 0.99)	0.035
Ethnicity					
Indigenous	3663 (5.50)	1.17 (1.00, 1.36)	0.043	1.79 (1.41, 2.28)	0.000
Ecuadorian	54,160 (81.32)	Reference			
Other	4806 (7.22)	0.91 (0.78, 1.05)	0.204	1.02 (0.87, 1.22)	0.768
Level of Education					
No formal education	1925 (2.89)	Reference			
Primary/Basic	28,724 (43.13)	1.00 (0.79, 1.25)	0.969	1.03 (0.81, 1.30)	0.834
Secondary and above	13,031 (19.57)	0.85 (0.67, 1.07)	0.165	1.03 (0.80, 1.32)	0.808
Child aged <20 years	6334 (9.51)	1.53 (1.20, 1.94)	0.001	2.68 (1.99, 3.60)	<0.001
Place of Death					
Public hospital	20,028 (30.07)	Reference			
Private hospital	7067 (10.61)	1.25 (1.13, 1.38)	<0.001	1.15 (0.99, 1.26)	0.080
Home	31,848 (47.82)	0.45 (0.41, 0.49)	<0.001	0.41 (0.37, 0.46)	<0.001
Other/No information	7655 (11.49)	0.28 (0.23, 0.33)	<0.001	0.34 (0.28, 0.42)	<0.001
Climate Region of Death					
Coastal and Insular	34,133 (51.25)	Reference			
Sierra	30,220 (45.38)	1.05 (0.98, 1.13)	0.180	0.94 (0.85, 1.03)	0.158
Amazon	2245 (3.37)	0.62 (0.47, 0.79)	<0.001	0.59 (0.42, 0.81)	0.001

Data are missing *N* (%) Age group 22 (0.03), Ethnicity 3969 (5.96), Level of Education 16642 (24.99).

Table S11: Association of sociodemographic risk factors and lower respiratory infection (LRI) mortality in Ecuador, 2016. Unadjusted odds ratio (OR) and adjusted odds ratio (aOR), 95% confidence intervals (CI) and *p* values are shown. Multivariable model includes sex, age group, area of residence, ethnicity, level of education, place of death and climate region of death. Associations with LRI mortality are compared with those for deaths from other causes.

		**Unadjusted 2016**		**Adjusted 2016**	
	***N* (%)**	**OR (95% CI)**	***p* value**	**OR (95% CI)**	***p* value**
Sex					
Male	37,716 (54.78)	Reference			
Female	31,132 (45.22)	1.09 (1.02, 1.17)	0.015	0.99 (0.92, 1.07)	0.877
Age Group (years)					
0–4	2683 (3.90)	3.27 (2.82, 3.80)	<0.001	1.71 (1.38, 2.13)	<0.001
5–69	30,182 (43.84)	Reference			
≥70	35,976 (52.25)	2.04 (1.89, 2.21)	<0.001	2.42 (2.20, 2.67)	<0.001
Area of Residence					
Urban	53,086 (77.11)	Reference			
Rural	15,762 (22.89)	0.87 (0.80, 0.95)	0.003	0.96 (0.87, 1.05)	0.367
Ethnicity					
Indigenous	3674 (5.34)	1.01 (0.87, 1.19)	0.858	1.19 (1.00, 1.42)	0.046
Ecuadorian	56,521 (82.10)	Reference			
Other	5061 (7.35)	0.84 (0.73, 0.97)	0.021	0.88 (0.76, 1.03)	0.113
Level of Education					
No formal education	15,184 (22.05)	Reference			
Primary/Basic	30,139 (43.78)	0.88 (0.80, 0.97)	0.001	0.88 (0.80, 0.98)	0.015
Secondary and above	13,954 (20.27)	0.79 0.71, 0.88)	<0.001	0.89 (0.79, 1.01)	0.083
Child aged <20 years	6341 (9.21)	1.40 (1.24, 1.58)	<0.001	1.97 (1.60, 2.41)	<0.001
Place of Death					
Public hospital	23,368 (33.94)	Reference			
Private hospital	6409 (9.31)	1.18 (1.06, 1.31)	0.002	1.12 (1.00, 1.26)	0.058
Home	33,791 (49.08)	0.47 (0.43, 0.51)	<0.001	0.42 (0.39, 0.46)	<0.001
Other/No information	5280 (7.67)	0.15 (0.11, 0.19)	<0.001	0.19 (0.14, 0.25)	<0.001
Climate Region of Death					
Coastal and Insular	35,666 (51.80)	Reference			
Sierra	30,961 (45.01)	0.99 (0.92, 1.06)	0.768	0.95 (0.87, 1.03)	0.179
Amazon	2221 (3.23)	0.66 (0.51, 0.84)	<0.001	0.71 (0.54, 0.92)	0.011

Data are missing *N* (%) Age Group 7 (0.01), Ethnicity 3592 (5.22), Level of Education 3276 (4.69).

Table S12: Association of sociodemographic risk factors and lower respiratory infection (LRI) mortality in Ecuador, 2017. Unadjusted odds ratio (OR) and adjusted odds ratio (aOR), 95% confidence intervals (CI) and *p* values are shown. Multivariable model includes sex, age group, area of residence, ethnicity, level of education, place of death and climate region of death. Associations with LRI mortality are compared with those for deaths from other causes.

		**Unadjusted 2017**		**Adjusted 2017**	
	***N* (%)**	**OR (95% CI)**	***p* value**	**aOR (95% CI)**	***p* value**
Sex					
Male	38,953 (54.99)	Reference			
Female	31,888 (45.01)	1.13 (1.05, 1.21)	0.001	1.00 (0.93, 1.08)	0.957
Age Group (years)					
0–4	2,779 (3.92)	3.50 (3.02, 4.05)	<0.001	1.80 (1.43, 2.24)	<0.001
5–69	30,091 (42.48)	Reference			
≥70	37,954 (53.58)	2.39 (2.21, 2.59)	<0.001	2.71 (2.47, 2.97)	<0.001
Area of Residence					
Urban	54,329 (76.69)	Reference			
Rural	16,512 (23.31)	0.99 (0.91, 1.07)	0.731	1.07 (0.98, 1.17)	0.126
Ethnicity					
Indigenous	3783 (5.34)	1.15 (0.99, 1.33)	0.062	1.39 (1.19, 1.63)	<0.001
Ecuadorian	58,173 (82.12)	Reference			
Other	5061 (7.07)	0.87 (0.76, 1.00)	0.056	0.93 (0.81, 1.08)	0.336
Level of Education					
No formal education	37,271 (52.61)	Reference			
Primary/Basic	15,043 (21.23)	0.86 (0.79, 0.94)	0.001	0.97 (0.88, 1.06)	0.466
Secondary and above	12,039 (16.99)	0.78 (0.70, 0.86)	<0.001	0.87 (0.77, 0.97)	0.015
Child aged <20 years	6320 (8.92)	1.30 (1.17, 1.45)	<0.001	2.03 (1.67, 2.46)	<0.001
Place of Death					
Public hospital	23,449 (33.10)	Reference			
Private hospital	8765 (12.37)	1.03 (0.94, 1.13)	0.535	0.95 (0.86, 1.06)	0.375
Home	33, 499 (47.29)	0.47 (0.43, 0.50)	<0.001	0.41 (0.38, 0.45)	<0.001
Other/No information	5128 (7.24)	0.13 (0.10, 0.17)	<0.001	0.17 (0.12, 0.22)	<0.001
Climate Region of Death					
Coastal and Insular	36,419 (51.41)	Reference			
Sierra	32,047 (45.24)	1.05 (0.98, 1.13)	0.136	0.98 (0.91, 1.06)	0.676
Amazon	2375 (3.35)	0.44 (0.34, 0.59)	0.000	0.45 (0.34, 0.60)	<0.001

Data are missing *N* (%) Age Group 17 (0.02), Ethnicity 3874 (5.47), Level of Education 195 (0.28).

Table S13: Association of sociodemographic risk factors and lower respiratory infection (LRI) mortality in Ecuador, 2018. Unadjusted odds ratio (OR) and adjusted odds ratio (aOR), 95% confidence intervals (CI) and *p* values are shown. Multivariable model includes sex, age group, area of residence, ethnicity, level of education, place of death and climate region of death. Associations with LRI mortality are compared with those for deaths from other causes.

		**Unadjusted 2018**		**Adjusted 2018**	
	***N* (%)**	**OR (95% CI)**	***p* value**	**OR (95% CI)**	***p* value**
Sex					
Male	40,120 (55.12)	Reference			
Female	32,669 (44.88)	1.17 (1.09, 1.25)	<0.001	1.04 (0.97, 1.11)	0.321
Age Group (years)					
0–4	2818 (3.87)	2.51 (2.17, 2.91)	<0.001	1.51 (1.21, 1.88)	<0.001
5–69	31,170 (42.82)	Reference			
≥70	38,741 (53.22)	1.85 (1.73, 1.99)	<0.001	2.03 (1.86, 2.21)	<0.001
Area of Residence					
Urban	55,756 (76.60)	Reference			
Rural	17,033 (23.40)	0.93 (0.86, 1.01)	0.077	1.01 (0.92, 1.10)	0.888
Ethnicity					
Indigenous	3715 (5.10)	0.96 (0.82, 1.11)	0.548	1.03 (0.87, 1.22)	0.733
Ecuadorian	59,823 (8.19)	Reference			
Other	4981 (6.84)	0.65 (0.56, 0.76)	<0.001	0.72 (0.61, 0.85)	<0.001
Level of Education					
No formal education	13,155 (18.07)	Reference			
Primary/Basic	33,352 (45.82)	0.85 (0.78, 0.93)	<0.001	0.85 (0.77, 0.93)	0.001
Secondary and above	15,572 (21.39)	0.78 (0.71, 0.87)	<0.001	0.89 (0.79, 1.00)	0.042
Child aged <20 years	6435 (8.84)	1.10 (0.98, 1.25)	0.111	1.31 (1.07, 1.60)	0.008
Place of Death					
Public hospital	26,052 (35.79)	Reference			
Private hospital	8586 (11.80)	0.89 (0.81, 0.97)	0.011	0.86 (0.78, 0.95)	0.003
Home	33,529 (46.06)	0.39 (0.36, 0.42)	<0.001	0.34 (0.31, 0.37)	<0.001
Other/No information	4622 (6.35)	0.11 (0.08, 0.148)	<0.001	0.13 (0.09, 0.18)	<0.001
Climate Region of Death					
Coastal and Insular	36,455 (50.08)	Reference			
Sierra	33,752 (46.37)	1.25 (1.17, 1.34)	<0.001	1.15(1.06, 1.23)	<0.001
Amazon	2582 (3.55)	0.68 (0.55, 0.85)	0.001	0.76 (0.60, 0.96)	0.022

Data are missing *N* (%) Age Group 60 (0.08), Ethnicity 4270 (5.87), Level of Education 4319 (5.93).

Table S14: Association of sociodemographic risk factors and lower respiratory infection (LRI) mortality in Ecuador, 2019. Unadjusted odds ratio (OR) and adjusted odds ratio (aOR), 95% confidence intervals (CI) and *p* values are shown. Multivariable model includes sex, age group, area of residence, ethnicity, level of education, place of death and climate region of death. Associations with LRI mortality are compared with those for deaths from other causes.

		**Unadjusted 2019**		**Adjusted 2019**	
	***N* (%)**	**OR (95% CI)**	***p* value**	**OR (95% CI)**	***p* value**
Sex					
Male	41,722 (55.37)	Reference			
Female	33,633 (44.63)	1.20 (1.12, 1.28)	<0.001	1.08 (1.01, 1.16)	0.031
Age group (years)					
0–4	2725 (3.62)	3.11 (2.68, 3.61)	<0.001	1.28 (1.04, 1.57)	0.021
5–69	32,295 (42.86)	Reference			
≥70	40,208 (53.36)	2.31 (2.14, 2.49)	<0.001	2.72 (2.49, 2.98)	<0.001
Area of Residence					
Urban	57,864 (76.79)	Reference			
Rural	17,491 (23.21)	0.97 (0.90, 1.05)	0.518	1.04 (0.96, 1.14)	0.321
Ethnicity					
Indigenous	3978 (5.28)	1.14 (0.99, 1.30)	0.071	1.35 (1.16, 1.57)	<0.001
Ecuadorian	61,628 (81.78)	Reference			
Other	5080 (6.74)	0.83 (0.72, 0.96)	0.009	0.91 (0.79, 1.06)	0.221
Level of Education					
No formal education	32,138 (42.65)	Reference			
Primary/Basic	21,200 (28.13)	0.81 (0.74, 0.88)	<0.001	0.93 (0.85, 1.01)	0.081
Secondary and above	15,470 (20.53)	0.83 (0.76, 0.91)	<0.001	1.00 (0.90, 1.11)	0.984
Child aged <20 years	6337 (8.41)	1.29 (1.16, 1.44)	<0.001	2.33 (1.96, 2.78)	<0.001
Place of Death					
Public hospital	27,158 (36.04)	Reference			
Private hospital	8767 (11.63)	1.01 (0.92, 1.11)	0.783	1.03 (0.93, 1.14)	0.538
Home	34,673 (46.01)	0.49 (0.45, 0.52)	<0.001	0.44 (0.40, 0.47)	<0.001
Other/No information	4757 (6.31)	0.14 (0.11, 0.19)	<0.001	0.18 (0.13, 0.25)	<0.001
Climate Region of Death					
Coastal and Insular	38,479 (51.06)	Reference			
Sierra	34,179 (45.36)	1.11 (1.04, 1.18)	0.002	0.99 (0.92, 1.06)	0.769
Amazon	2697 (3.58)	0.77 (0.63, 0.94)	0.010	0.68 (0.54, 0.86)	0.001

Data are missing *N* (%) Age Group 127 (0.17), Ethnicity 4669 (6.20), Level of Education 238 (0.32).

Table S15: Association of sociodemographic risk factors and lower respiratory infection (LRI) mortality in Ecuador, 2020. Unadjusted odds ratio (OR) and adjusted odds ratio (aOR), 95% confidence intervals (CI) and *p* values are shown. Multivariable model includes sex, age group, area of residence, ethnicity, level of education, place of death and climate region of death. Associations with LRI mortality are compared with those for deaths from other causes.

		**Unadjusted 2020**		**Adjusted 2020**	
	***N* (%)**	**OR (95% CI)**	***p* value**	**OR (95% CI)**	***p* value**
Sex					
Male	68,914 (58.89)	Reference			
Female	48,116 (41.11)	0.70 (0.68, 0.72)	<0.001	0.70 (0.68, 0.73)	<0.001
Age Group (years)					
0–4	2098 (1.79)	0.24 (0.20, 0.28)	<0.001	0.90 (0.73, 1.12)	0.360
5–69	51,845 (44.30)	Reference			
≥70	63,065 (53.89)	0.75 (0.73, 0.77)	<0.001	0.98 (0.95, 1.02)	0.352
Area of Residence					
Urban	92,744 (79.25)	Reference			
Rural	24,286 (20.75)	0.66 (0.64, 0.69)	<0.001	0.88 (0.84, 0.92)	<0.001
Ethnicity					
Indigenous	4815 (4.11)	0.51 (0.47, 0.56)	<0.001	0.93 (0.84, 1.02)	0.119
Ecuadorian	95,623 (81.71)	Reference			
Other	6821 (5.83)	0.49 (0.46, 0.52)	<0.001	0.59 (0.55, 0.64)	<0.001
Level of Education					
No formal education	18,953 (16.19)	Reference			
Primary/Basic	55,007 (47.00)	1.96 (1.87, 2.04)	<0.001	1.40 (1.33, 1.47)	<0.001
Secondary and above	28,554 (24.40)	2.00 (1.91, 2.10)	<0.001	1.25 (1.18, 1.32)	<0.001
Child aged <20 years	5288 (4.52)	0.47 (0.42, 0.52)	<0.001	0.20 (0.17, 0.23)	<0.001
Place of Death					
Public hospital	42,626 (36.42)	Reference			
Private hospital	10,301 (8.80)	0.31 (0.30, 0.33)	<0.001	0.30 (0.28, 0.31)	<0.001
Home	58,149 (49.69)	0.10 (0.09, 0.10)	<0.001	0.10 (0.10, 0.10)	<0.001
Other/No information	5954 (5.09)	0.11 (0.10, 0.12)	<0.001	0.04 (0.03, 0.05)	<0.001
Climate Region of Death					
Coastal and Insular	65,623 (56.07)	Reference			
Sierra	47,781 (40.83)	1.08 (1.05, 1.11)	<0.001	1.05 (1.05, 1.13)	<0.001
Amazon	3629 (3.10)	0.85 (0.79, 0.92)	<0.001	0.87 (0.87, 1.05)	0.374

Data are missing *N* (%) Age Group 22 (0.02), Ethnicity 9771 (8.35), Level of Education 9271 (7.92).

Table S16: Association of sociodemographic risk factors and lower respiratory infection (LRI) mortality in Ecuador, 2021. Unadjusted odds ratio (OR) and adjusted odds ratio (aOR), 95% confidence intervals (CI) and *p* values are shown. Multivariable model includes sex, age group, area of residence, ethnicity, level of education, place of death and climate region of death. Associations with LRI mortality are compared with those for deaths from other causes.

		**Unadjusted 2021**		**Adjusted 2021**	
	***N* (%)**	**OR (95% CI)**	***p* value**	**OR (95% CI)**	***p* value**
Sex					
Male	61,426 (57.06)	Reference			
Female	46,222 (42.94)	0.84 (0.82, 0.87)	<0.001	0.84 (0.81, 0.87)	<0.001
Age Group (years)					
0–4	2153 (2.00)	0.25 (0.21, 0.29)	<0.001	0.88 (0.70, 1.09)	0.238
5–69	49,513 (46.00)	Reference			
≥70	55,932 (51.96)	0.84 (0.81, 0.86)	<0.001	1.03 (0.99, 1.07)	0.102
Area of Residence					
Urban	83,113 (77.21)	Reference			
Rural	24,535 (22.79)	0.70 (0.68, 0.73)	<0.001	0.90 (0.86, 0.94)	<0.001
Ethnicity					
Indigenous	4562 (4.24)	0.39 (0.36, 0.43)	<0.001	0.73 (0.66, 0.82)	<0.001
Ecuadorian	92,653 (86.07)	Reference			
Other	5885 (5.47)	0.44 (0.40, 0.47)	<0.001	0.62 (0.57, 0.68)	<0.001
Level of Education					
No formal education	18,012 (16.73)	Reference			
Primary/Basic	46,173 (42.89)	2.61 (2.49, 2.73)	<0.001	1.55 (1.47, 1.64)	<0.001
Secondary and above	32,814 (30.48)	1.56 (1.48, 1.64)	<0.001	1.35 (1.28, 1.43)	<0.001
Child aged <20 years	5774 (5.36)	0.43 (0.38, 0.48)	<0.001	0.24 (0.20, 0.28)	<0.001
Place of Death					
Public hospital	41,537 (38.42)	Reference			
Private hospital	11,979 (11.13)	0.47 (0.45, 0.49)	<0.001	0.46 (0.44, 0.48)	<0.001
Home	48,120 (44.70)	0.09 (0.09, 0.09)	<0.001	0.09 (0.09, 0.10)	<0.001
Other/No information	6192 (5.75)	0.04 (0.03, 0.05)	<0.001	0.03 (0.02, 0.03)	<0.001
Climate Region of Death					
Coastal and Insular	54,945 (51.04)	Reference			
Sierra	48,871 (45.40)	1.30 (1.27, 1.34)	<0.001	1.46 (1.41, 1.51)	<0.001
Amazon	3832 (3.56)	0.82 (0.75, 0.89)	<0.001	1.08 (0.98, 1.19)	0.135

Data are missing *N* (%) Age Group 50 (0.05), Ethnicity 4548 (4.22), Level of Education 4925 (4.58).

Table S17: Association of sociodemographic risk factors and lower respiratory infection (LRI) mortality in Ecuador, 2022. Unadjusted odds ratio (OR) and adjusted odds ratio (aOR), 95% confidence intervals (CI) and *p* values are shown. Multivariable model includes sex, age group, area of residence, ethnicity, level of education, place of death and climate region of death. The association for LRI mortality is compared with that to deaths from other causes.

		**Unadjusted 2022**		**Adjusted 2022**	
	***N* (%)**	**OR (95% CI)**	***p* value**	**OR (95% CI)**	***p* value**
Sex					
Male	51,942 (56.49)	Reference			
Female	40,012 (43.51)	1.02 (0.97, 1.07)	0.434	0.90 (0.86, 0.95)	<0.001
Age Group (years)					
0–4	2249 (2.45)	1.92 (1.66, 2.22)	<0.001	1.38 (1.12, 1.70)	0.003
5–69	40,950 (44.53)	Reference			
≥70	48,630 (52.89)	1.92 (1.82, 2.02)	<0.001	2.32 (2.18, 2.46)	<0.001
Area of Residence					
Urban	70,081 (76.21)	Reference			
Rural	21,873 (23.79)	0.78 (0.73, 0.82)	<0.001	0.96 (0.90, 1.03)	0.285
Ethnicity					
Indigenous	4300 (4.68)	0.77 (0.68, 0.87)	<0.001	1.21 (1.05, 1.39)	0.007
Ecuadorian	77,698 (84.50)	Reference			
Other	5819 (6.33)	0.58 (0.510.65)	<0.001	0.70 (0.62, 0.80)	<0.001
Level of Education					
No formal education	15,619 (16.99)	Reference			
Primary/Basic	42,402 (46.11)	1.17 (1.09, 1.26)	<0.001	1.02 (0.94, 1.10)	0.651
Secondary and above	22,253 (24.20)	1.22 (1.13, 1.32)	<0.001	1.16 (1.06, 1.27)	0.001
Child aged <20 years	6244 (6.79)	1.00 (0.89, 1.13)	0.958	1.02 (0.86, 1.12)	0.826
Place of Death					
Public hospital	30,011 (32.64)	Reference			
Private hospital	9704 (10.55)	0.66 (0.61, 0.70)	<0.001	0.64 (0.60, 0.69)	<0.001
Home	44,160 (48.02)	0.18 (0.17, 0.19)	<0.001	0.16 (0.15, 0.17)	<0.001
Other/No information	8079 (8.79)	0.06 (0.05, 0.08)	<0.001	0.05 (0.04, 0.07)	<0.001
Climate Region of Death					
Coastal and Insular	49,277 (53.59)	Reference			
Sierra	39,341(42.78)	0.99 (0.94, 1.04)	0.708	0.94 (0.89, 0.99)	0.023
Amazon	3363 (3.70)	0.77 (0.67, 0.89)	<0.001	0.80 (0.69, 0.94)	0.008

Data are missing *N* (%) Age Group 125 (0.14), Ethnicity 4137 (4.50), Level of Education 5507 (6.06).
